# Ubiquitin‐Specific Protease 22 Plays a Key Role in Increasing Extracellular Vesicle Secretion and Regulating Cell Motility of Lung Adenocarcinoma

**DOI:** 10.1002/advs.202405731

**Published:** 2024-08-05

**Authors:** Fang Zhen, Yue Sun, Hongyi Wang, Wei Liu, Xiao Liang, Yaru Wang, Qi Wang, Jing Hu

**Affiliations:** ^1^ Department of Breast Medical Oncology Harbin Medical University Cancer Hospital Harbin Medical University No. 150 Haping Road Harbin Heilongjiang 150040 China; ^2^ Key laboratory of Preservation of Human Genetic Resources and Disease Control in China (Harbin Medical University) Ministry of Education Harbin Heilongjiang 150081 China; ^3^ Department of Medicinal Chemistry and Natural Medicinal Chemistry College of Pharmacy Harbin Medical University No. 157 Baojian Road Harbin Heilongjiang 150081 China

**Keywords:** extracellular vesicles, invadopodia, lung adenocarcinoma, tumor cell metastasis, ubiquitin‐specific protease 22

## Abstract

Tumor‐derived extracellular vesicles (EVs) are potential biomarkers for tumors, but their reliable molecular targets have not been identified. The previous study confirms that ubiquitin‐specific protease 22 (USP22) promotes lung adenocarcinoma (LUAD) metastasis in vivo and in vitro. Moreover, USP22 regulates endocytosis of tumor cells and localizes to late endosomes. However, the role of USP22 in the secretion of tumor cell‐derived EVs remains unknown. In this study, it demonstrates that USP22 increases the secretion of tumor cell‐derived EVs and accelerates their migration and invasion, invadopodia formation, and angiogenesis via EV transfer. USP22 enhances EV secretion by upregulating myosin IB (MYO1B). This study further discovers that USP22 activated the SRC signaling pathway by upregulating the molecule KDEL endoplasmic reticulum protein retention receptor 1 (KDELR1), thereby contributing to LUAD cell progression. The study provides novel insights into the role of USP22 in EV secretion and cell motility regulation in LUAD.

## Graphical abstract

USP22 increases the secretion of tumor cell‐derived EVs. USP22‐mediated EV secretion contributes to invadopodia formation, which are specific and critical docking and secretion sites for MVBs, thus forming a positive feedback loop to promote tumor metastasis. The study provides a novel insight into the role of USP22 in EVs secretion and regulating cell motility in LUAD.

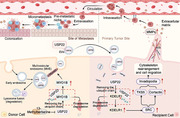

## 1 Introduction

Lung cancer is the leading cause of morbidity and mortality among malignant tumors worldwide, with an estimated 1.8 million deaths.^[^ ^1^ ^]^ The most common subtype is lung adenocarcinoma (LUAD). Unfortunately, most patients with LUAD have distant metastasis at the time of diagnosis, and the 5‐year overall survival rate is less than 20%, indicating a poor prognosis.^[^ ^2^ ^]^ Therefore, it is important to investigate the mechanisms underlying LUAD progression and identify valuable biomarkers and therapeutic targets.

Extracellular vesicles (EVs), including exosomes and ectosomes, are lipid bilayer nanoparticles released by most cell types. EVs are carriers of functional biomolecules, including nucleic acids, proteins, and lipids.^[^ ^3^ ^]^ EVs play a crucial role in local and distant cellular communication between tumor cells and the surrounding tumor microenvironment (TME). Tumor cell‐derived EVs can contribute to multiple physiological and pathological processes, such as angiogenesis, extracellular matrix (ECM) remodeling, immunosuppression, and other biological effects.^[^ ^4^ ^]^ Tumor tissues release large amounts of “functional molecules” containing EVs into the ECM to meet the high demand for nutrients and energy, which serve as “seeds” for tumor cell reproduction. EVs can alter the structure and function of target organs and tissues in the TME, leading to tumor metastasis.^[^ ^5^ ^]^ These functional molecules containing EVs are valuable indicators of tumor progression and provide methods for early tumor diagnosis.^[^ ^6^ ^]^ EVs contribute to tumor progression and metastasis during LUAD development.^[^ ^7^, ^8^, ^9^ ^]^ However, the mechanisms underlying EV secretion remain unclear.

Ubiquitin‐specific protease 22 (USP22) is a deubiquitinating enzyme belonging to the USP family. USP22 contributes to histone H2A/H2B deubiquitination and activates transcription factors during tumor progression.^[^ ^10^ ^]^ USP22 participates in cell cycle progression by stabilizing Cyclin B1,^[^ ^11^ ^]^ Cyclin D1,^[^ ^12^ ^]^ and sirtuin 1.^[^ ^13^ ^]^ USP22 reduces SPI1 degradation through deubiquitination, which enhances programmed death‐ligand 1 (PD‐L1) transcription.^[^ ^14^ ^]^ USP22 promotes de novo synthesis of fatty acids and tumorigenesis by deubiquitinating peroxisome proliferator‐activated receptor gamma (PPARγ).^[^ ^15^ ^]^ USP22 promotes cancer cell survival after DNA damage by maintaining XPC stability.^[^ ^16^ ^]^ Our previous study found that USP22 promotes cell proliferation, migration, and invasion and contributes to epidermal growth factor‐tyrosine kinase inhibitor (EGFR‐TKI) resistance by preventing EGFR degradation in EGFR‐mutant LUAD cells.^[^ ^17^ ^]^ There are fewer studies on ubiquitination and EVs. It has been suggested that ubiquitination may play an important role in the sorting process of EV proteins.^[^ ^18^ ^]^ However, the roles of USP22 in EV secretion and tumor cell‐derived EVs have not yet been identified.

In this study, we focused our attention on USP22 regulating the secretion of EVs and cell motility. Our study suggests that USP22 is a novel expected to be a new therapeutic target for LUAD.

## 2 Results

### 2.1 USP22 Regulates the Secretion of EVs

**Figure 1:**
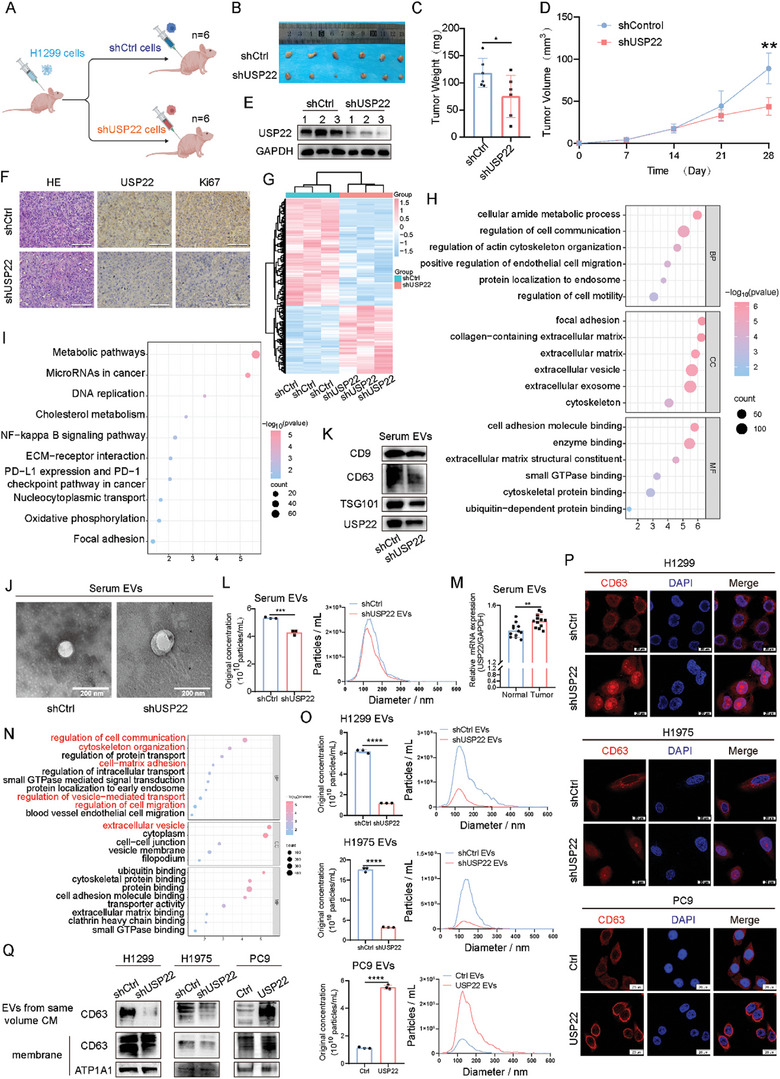
USP22 regulates the secretion of EVs. A) Schematic representation of the experimental design of the xenograft model (n = 6 mice per group). B–D) Tumor images (B), growth weights (C), and proliferation curves (D). E) USP22 was analyzed using western blotting of the xenograft tumors. F) HE, and IHC staining of Ki67 and USP22. Scale bars, 25 µm. G) Heatmap of the differences between mice with USP22‐Ctrl and USP22 knockdown after proteomic sequencing. H,I) GO (H) and KEGG (I) analysis based on the differential genes of the xenograft tumors. J,K) Characterization of serum‐derived EVs using TEM (J), western blotting analysis (K) of CD9, CD63, TSG101 (EV‐derived protein markers), and USP22. Scale bars, 200 nm. L) NTA analysis of the effect of USP22 on EV secretion in vivo. M) Relative mRNA expression of USP22 in serum‐derived EVs of healthy individuals and patients with LUAD detected using qRT‐PCR (n = 12). N) GO analysis of differentially expressed proteins in USP22 knockdown and control H1299 cells. O) NTA results of EVs revealing the total number of particles isolated from each group. P) Confocal microscopy analysis of CD63 in LUAD lines. Scale bars, 20 µm. Q CD63 expression of EVs isolated from the same culture media (CM) and cell membrane proteins were tested using western blotting. Data are presented as mean ± SD. Statistical significance was determined by Student's *t*‐test and ANOVA test. ^*^ *p* < 0.05, ^**^ *p* < 0.01, ^***^ *p* < 0.001, ^****^ *p* < 0.0001.

Our previous study confirmed that USP22 is strongly associated with a poor prognosis of LUAD. We constructed cell lines with USP22 knockdown (H1299 and H1975) and overexpression (PC9) using lentiviral vectors (Figure S1A,B, Supporting Information). To explore the role of USP22 in LUAD progression in vivo, we transplanted cells with USP22 knockdown and control H1299 cells into nude mice subcutaneously (**Figure** **1**A). USP22 knockdown resulted in smaller tumor volumes and weights compared with those in mice implanted with control shRNA‐infected cells (Figure 1B–D). First, we used western blotting to verify the expression of USP22 in the tumor tissues of each group (Figure 1E). Thereafter, we performed hematoxylin and eosin (HE) and immunohistochemical (IHC) staining. IHC staining showed that the expression of USP22 and Ki67 was significantly reduced in the tumor tissues of mice in the USP22 knockdown group than in the control group (Figure 1F). We performed proteomic sequencing of tumor tissues from each group, and the results of differential gene‐based gene ontology (GO) and Kyoto Encyclopedia of Genes and Genomes (KEGG) analyses showed that USP22 was closely associated with biological functions, such as cell communication, cell motility, EV secretion, ECM‐receptor interaction, and focal adhesion (Figure 1G–I). Therefore, we speculated that USP22 could influence malignant tumor phenotypic transformation by regulating EV secretion.

To explore whether USP22 regulates EV secretion in vivo, we isolated EVs from mice serum by differential ultracentrifugation and identified them using transmission electron microscopy (TEM), western blotting, and nanoparticle tracking analysis (NTA). We observed that the mean size of EVs isolated from the same volume of serum was not affected in mice with USP22 knockdown compared with that in the controls (Figure 1J), whereas western blotting analysis revealed reduced expression of EV‐derived protein markers (CD9, CD63, and TSG101) in the serum‐derived EVs of mice with USP22 knockdown, as well as reduced USP22 expression (Figure 1K). According to NTA results, fewer EVs were isolated from mice with USP22 knockdown (Figure 1L). Finally, we quantified USP22 expression in serum‐derived EVs from patients with LUAD and healthy individuals using quantitative reverse transcription polymerase chain reaction (qRT‐PCR) and found that USP22 expression was significantly higher in patients with LUAD than in healthy individuals (Figure 1M).

To verify the mechanisms by which USP22 promotes EV secretion, we selected the USP22 knockdown cell line, H1299, for proteomic sequencing. GO enrichment analysis showed that the differentially expressed genes (DEGs) were closely related to pathways, such as cell communication, cell migration, EV secretion, and cell‐matrix adhesion (Figure 1N). We isolated EVs from cell supernatants by differential ultracentrifugation, and they were identified using TEM and western blotting. TEM revealed that secreted EVs have a diameter of ≈50–140 nm (Figure S1C, Supporting Information). Western blotting analysis showed the presence of EV protein markers (CD9, CD63, and TSG101) and the absence of Calnexin in the EVs (Figure S1D, Supporting Information). PKH26‐labeled EVs were incubated with LUAD cells, and red signals were observed in recipient cells after co‐culturing with LUAD cells for 12 h. This demonstrated that tumor cell‐derived EVs were successfully absorbed by the recipient LUAD cells (Figure S1E, Supporting Information). NTA results showed a decrease in EV secretion after USP22 knockdown and an increase in EV secretion in cells with USP22 overexpression (Figure 1O). As identified by proteomics, USP22 knockdown reduced EV secretion from LUAD cells compared with the control.

Our previous study confirmed that USP22 is localized to late endosomes,^[^ ^17^ ^]^ known as multivesicular bodies (MVBs). Therefore, we explored the influence of USP22 on MVB transport. CD63 is typically used as a marker for MVBs.^[^ ^19^ ^]^ We then verified the location of CD63 using immunofluorescence (IF). CD63 was closer to the nucleus of cells with USP22 knockdown, whereas CD63 was closer to the plasma membrane after USP22 overexpression (Figure 1P). Western blotting showed that USP22 overexpression increased the secretion of EV‐containing CD63 and that the expression level of CD63 at the plasma membrane increased after USP22 overexpression (Figure 1Q).

In conclusion, these results strongly support the role of USP22 in regulating EV secretion in vivo and in vitro.

### 2.2 USP22 Enhances MVB Transport to the Plasma Membrane by Inhibiting the Ubiquitin‐Proteasomal Degradation of MYO1B

**Figure 2:**
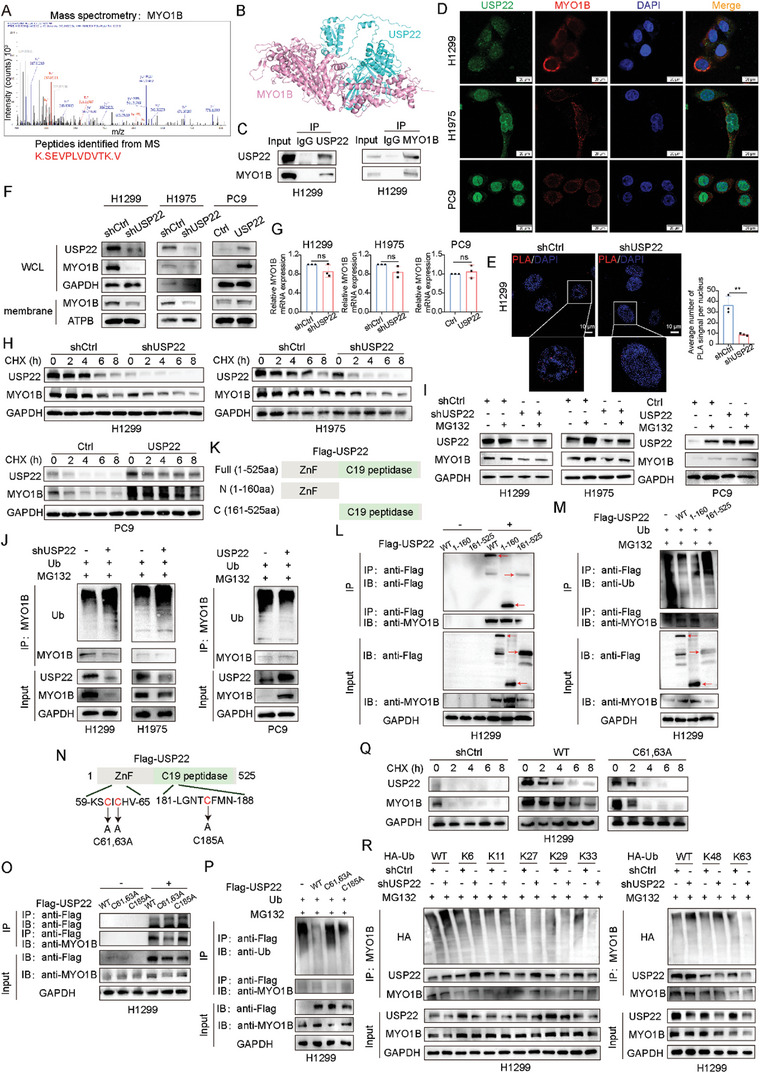
USP22 regulates EV secretion from LUAD cell lines via stabilizing MYO1B. A) MS identification of MYO1B as a potential USP22 interacting protein in H1299 cells. B) Visualization of USP22‐MYO1B binding. C) Co‐IP assays revealing the interaction between USP22 and MYO1B in H1299 cells. D) IF imaging shows the colocalization of USP22 and MYO1B in LUAD cells. Scale bars, 20 µm. E) PLA assay was performed in shCtrl and shUSP22 H1299 cells: each PLA signal represents a molecular interaction between USP22 and MYO1B. Scale bars, 10 µm. F) Western blotting of MYO1B protein expression in LUAD cell lines with USP22 knockdown and overexpression. G) qRT‐PCR of MYO1B expression in LUAD cell lines with USP22 knockdown and overexpression. H) Western blotting of MYO1B expression treated with CHX (200 µg mL^−1^). I) Western blotting of MYO1B expression treated with MG132 (10 µm) for 6 h. J) Impact of USP22 knockdown and overexpression on MYO1B ubiquitination. K) Schematic representation of the structures of USP22 and its truncated mutants. L) MYO1B interactions with USP22 and its mutants. M) Determination of MYO1B ubiquitination via co‐IP and western blotting analysis of MYO1B using an anti‐ubiquitin antibody. N) Schematic representation of USP22 and its point mutants. O) MYO1B interactions with USP22 and its point mutants. P) USP22 wild type or point mutants were transfected to measure MYO1B ubiquitination in H1299 cells. Q) MYO1B and USP22 protein levels were detected in H1299 cells treated with CHX. R) H1299 cells were co‐transfected with HA‐Ub WT, or HA‐K6‐Ub (Lys6‐only), or HA‐K11‐Ub (Lys11‐only), or HA‐K27‐Ub (Lys27‐only), or HA‐K29‐Ub (Lys29‐only), or HA‐K33‐Ub (Lys33‐only), or HA‐K48‐Ub (Lys48‐only), or HA‐K63‐Ub (Lys63‐only) plasmids, and then the MYO1B ubiquitylation linkage was detected using IP analysis with anti‐HA antibody after treatment with MG132 for 6 h. Data are presented as mean ± SD. Statistical significance was determined by Student's *t*‐test. Not significant (ns), *p* > 0.05, ^*^ *p* < 0.05, ^**^ *p* < 0.01, ^***^ *p* < 0.001, ^****^ *p* < 0.0001.

EVs are contained within MVBs, which are transported along microtubules to the plasma membrane and secreted into the extracellular environment.^[^ ^20^, ^21^ ^]^ To determine the mechanism by which USP22 affects MVB transport, we performed mass spectrometry （MS) to identify potential binding proteins in H1299 cells. We focused on the myosin (MYO) protein family members, and MS results revealed that MYO1B was significantly enriched by the USP22 antibody compared with the control immunoglobulin G (IgG) (**Figure** **2**A). A previous MS study on HEK293T cells transfected with USP22 detected MYO1B as an interacting protein.^[^ ^15^ ^]^ MYO1B is a type of myosin and a motor protein involved in various biological processes, such as cell migration, cytoskeleton organization, and vesicle transport.^[^ ^22^ ^]^ Molecular docking simulations were performed to verify the interaction between USP22 and MYO1B (Figure 2B). Co‐IP experiments showed that USP22 interacted with MYO1B (Figure 2C). We found USP22 was localized in both nucleus and cytoplasm,^[^ ^23^, ^24^, ^25^ ^]^ and partially colocalized with MYO1B in the cytoplasm in LUAD cells using confocal fluorescence microscopy (Figure 2D). We used the Proximity ligation assay (PLA) to detect endogenous protein interactions. We used USP22 and MYO1B antibodies and confirmed that the two protein complexes interacted (in the 40 nm range), as shown by the PLA signal. We observed an increase in the PLA signal in shCtrl H1299 cells than in shUSP22 H1299 cells (Figure 2E). After identifying the interaction between USP22 and MYO1B, further verification was performed. We performed western blotting to detect the protein expression of MYO1B after USP22 knockdown and overexpression. The results suggested that the total protein expression level of MYO1B decreased after USP22 knockdown and that the protein expression level of MYO1B on the cell membrane decreased (Figure 2F). We examined the effect of USP22 on the mRNA expression level of MYO1B using qRT‐PCR. Neither the USP22 knockdown nor overexpression affected the mRNA expression of MYO1B (Figure 2G). These results suggest that USP22 does not affect the mRNA expression of MYO1B and that the regulatory effect of USP22 on MYO1B occurs at the post‐translational level.

We examined the effects of USP22 knockdown and overexpression on the stability of MYO1B following cycloheximide (CHX) treatment. USP22 knockdown markedly shortened the half‐life of MYO1B, while USP22 overexpression prolonged its half‐life (Figure 2H). Notably, the USP22 knockdown‐mediated decreased MYO1B expression was reversed by a proteasome inhibitor (MG132) (Figure 2I). Ubiquitination assays showed that USP22 knockdown resulted in higher polyubiquitination levels in H1299 and H1975 cell lines and that USP22 overexpression decreased MYO1B polyubiquitination levels in PC9 cell lines (Figure 2J). USP22 contains an N‐terminal zinc‐finger structural domain and a C19 ubiquitin‐specific peptidase structural domain. To identify the USP22 region that interacted with MYO1B, we generated two USP22 truncated mutants of H1299 cells (Figure 2K). The N‐terminal zinc finger domain of USP22 binds strongly to MYO1B, whereas the C‐terminal ubiquitin‐specific peptidase domain binds weakly to MYO1B (Figure 2L). In addition, the N‐terminal zinc finger domain of USP22 is involved in regulating MYO1B ubiquitination (Figure 2M). Moreover, we constructed H1299 cells with USP22 point mutants (Figure 2N). In line with the truncated mutant results, the C‐terminal ubiquitin‐specific peptidase domain of USP22 with a point mutation strongly binds to MYO1B, and the zinc finger‐containing N‐terminus with a point mutation binds weakly (Figure 2O). The N‐terminal of USP22, which contained a point mutation, did not affect MYO1B ubiquitination (Figure 2P). The N‐terminal of USP22 containing a point mutation did not affect the degradation of MYO1B following CHX treatment (Figure 2Q). Further analysis showed that USP22 efficiently cleaved MYO1B polyubiquitination linked to lys48 but had little effect on the ubiquitination of MYO1B linked to lys6, lys11, lys27, lys29, lys33, and lys63 (Figure 2R).

These results confirmed that USP22 regulates the intracellular transport of MVBs by inhibiting the ubiquitin‐proteasomal degradation of MYO1B and stabilizing MYO1B expression.

### 2.3 LUAD Cell‐Derived EVs Secrete USP22 to Promote Tumor Progression

**Figure 3:**
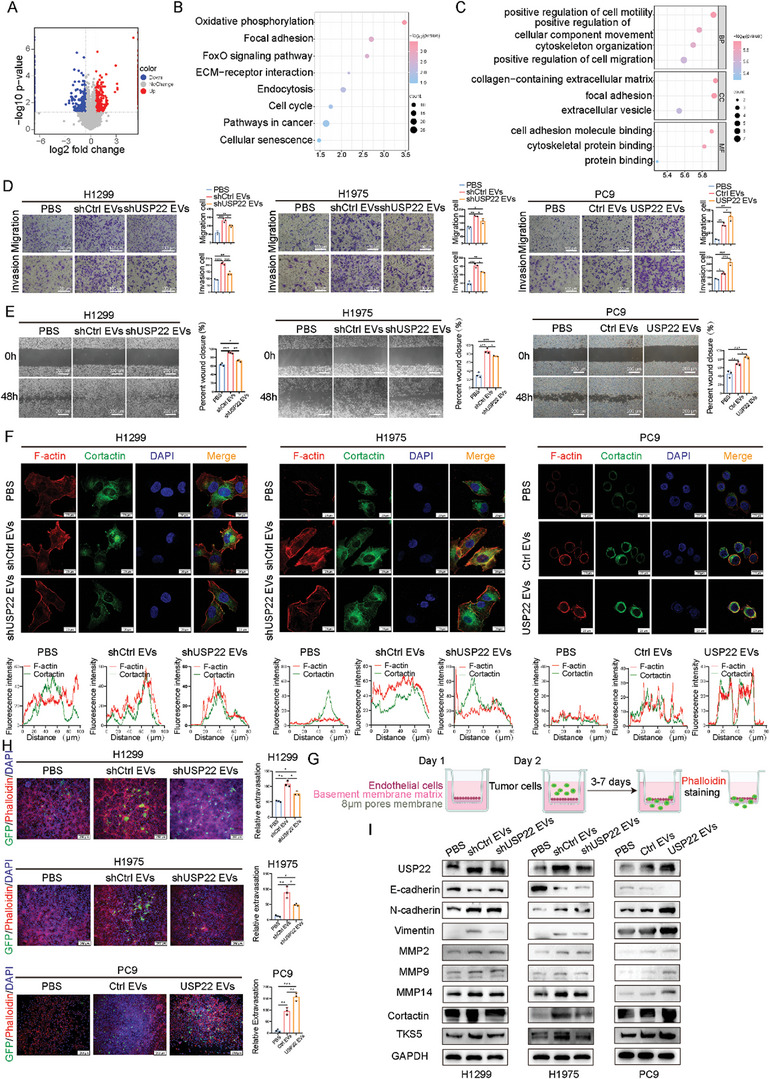
EV‐derived USP22 isolated from LUAD cells enhances tumor cell motility. A) Volcano plot of differentially expressed proteins of shCtrl EVs and shUSP22 EVs in H1299 cells obtained via proteomic analysis. B) KEGG enrichment analysis. C) GO enrichment analysis. D) Cell migration and invasion abilities of LUAD cells co‐cultured with EVs were examined using transwell assay. Scale bars, 100 µm. E) Cell migration ability of LUAD cells co‐cultured with EVs were examined via wound healing assay. Scale bars, 200 µm. F) LUADs treated with EVs were stained with invadopodia markers, cortactin and F‐actin (Phalloidin). Scale bars, 20 µm. G) In vitro metastasis model. H) Trans‐endothelial migration and intravasation/extravasation. Scale bars, 250 µm. I) Western blotting analysis of the effect of EVs on the expression of malignant proteins in LUAD cells. Data are presented as mean ± SD. Statistical significance was determined by ANOVA test. ^*^ *p* < 0.05, ^**^ *p* < 0.01, ^***^ *p* < 0.001, ^****^ *p* < 0.0001.

We focused on revealing the impact of USP22‐mediated EV secretion on tumor progression. We performed proteomic analysis of H1299 cell‐derived EVs to determine their proteomic landscape. Differential proteomic data analysis revealed 311 downregulated and 340 upregulated proteins between shCtrl and shUSP22 H1299 cell‐derived EVs (**Figure** **3**A). KEGG and GO enrichment analysis revealed that EV‐derived USP22 is mainly involved in regulating signaling pathways, such as cell motility, focal adhesion, and cytoskeleton organization (Figure 3B,C). Additionally, we performed transwell assays, and the results revealed that EV‐derived USP22 significantly induced the migration and invasion of LUAD cells (Figure 3D). The wound healing assay showed that the migration ability of LUAD cells was enhanced after co‐culturing them with EVs (Figure 3E). Previous studies have confirmed that invadopodia formation provides docking and secretion sites for exosomes and promotes tumor exosome secretion, thereby accelerating tumor progression.^[^ ^21^, ^26^ ^]^ We thereafter verified invadopodia formation using invadopodia markers (cortactin and F‐actin) after co‐culturing LUAD cells with EVs. These results indicated that EV‐derived USP22 markedly strengthened invadopodia formation, forming a positive feedback regulatory loop (Figure 3F). To further reveal the ability of EV‐derived USP22 to promote tumor metastasis, we constructed an in vitro metastasis model (Figure 3G). This system mimics the invasion of LUAD cells through the local basement membrane at the primary site, migration into or out of the vascular system (via trans‐endothelial migration of human umbilical endothelial cells (HUVECs)), and subsequent transversion of the basement membrane, followed by metastasis to distant sites.^[^ ^27^ ^]^ EV‐derived USP22 significantly promoted trans‐endothelial migration, extravasation, and subsequent metastasis of LUADs compared with the controls (Figure 3H). Moreover, LUAD cells treated with EVs showed increased expression of USP22, N‐cadherin, vimentin, matrix metallopeptidase （MMP）2, MMP9, MMP14, cortactin, and TKS5 but decreased expression of E‐cadherin (Figure 3I).

We investigated the effects of EVs on other biological functions of LUAD cells. We found that the proliferation and colony formation abilities of LUAD cells were significantly enhanced after they were co‐cultured with EVs compared with phosphate buffer saline (PBS) (Figure S2A–C, Supporting Information). We used migration and tube formation assays to investigate the function of EV‐derived USP22 in vascular endothelial cells. These results showed that treatment with USP22‐overexpressing EVs potentiated HUVECs migration during wound healing (Figure S2D, Supporting Information) and transwell assays (Figure S2E, Supporting Information) and the angiogenic ability of HUVECs in the tube formation assay (Figure S2F, Supporting Information).

**Figure 4:**
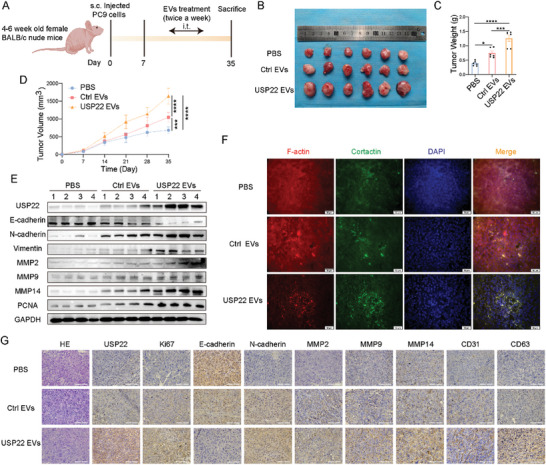
EV‐derived USP22 promotes tumor growth in xenograft tumors. A) Schematic diagram of the xenograft tumor model. (n = 6 mice per group). B–D) Tumor images (B), growth weights (C), and proliferation curves (D). E) Western blotting analysis of the expression of USP22, E‐cadherin, N‐cadherin, vimentin, MMP2, MMP9, MMP14, and PCNA. F) IF images of invadopodia in tumor tissues. G) HE and IHC staining of USP22, Ki67, E‐cadherin, N‐cadherin, MMP2, MMP9, MMP14, CD31, and CD63. Scale bars, 25 µm. Data are presented as mean ± SD. Statistical significance was determined by ANOVA test. ^*^ *p* < 0.05, ^**^ *p* < 0.01, ^***^ *p* < 0.001, ^****^ *p* < 0.0001.

LUAD mice xenograft tumor models were performed to investigate the effects of EV‐derived USP22 in vivo. EVs (Ctrl EVs and USP22 EVs) and PBS were injected into the center of the tumors twice a week, starting one week after the subcutaneous injection of PC9 cells (**Figure** **4**A). The results showed that the tumor volume in mice treated with EVs was larger than that in the controls, and the weight of the tumor also increased (Figure 4B–D). Western blotting was performed to determine the expression of USP22, E‐cadherin, N‐cadherin, vimentin, MMP2, MMP9, MMP14, and proliferative cell nuclear antigen (PCNA) (Figure 4E). We performed IF staining of the tumor tissues. These results indicate that EV‐derived USP22 enhanced invadopodia formation in vivo (Figure 4F). HE and IHC staining indicated that E‐cadherin expression was reduced, and N‐cadherin, MMP2, MMP9, MMP14, CD31, CD63, Ki67, and USP22 expression was increased in EV‐treated tumors compared with the controls (Figure 4G).

The above findings demonstrate that EV‐derived USP22 can be transported into LUAD cells and promote tumor development both in vivo and in vitro.

### 2.4 USP22 Interacts with KDELR1 and Stabilizes KDELR1 Expression via Deubiquitination

**Figure 5:**
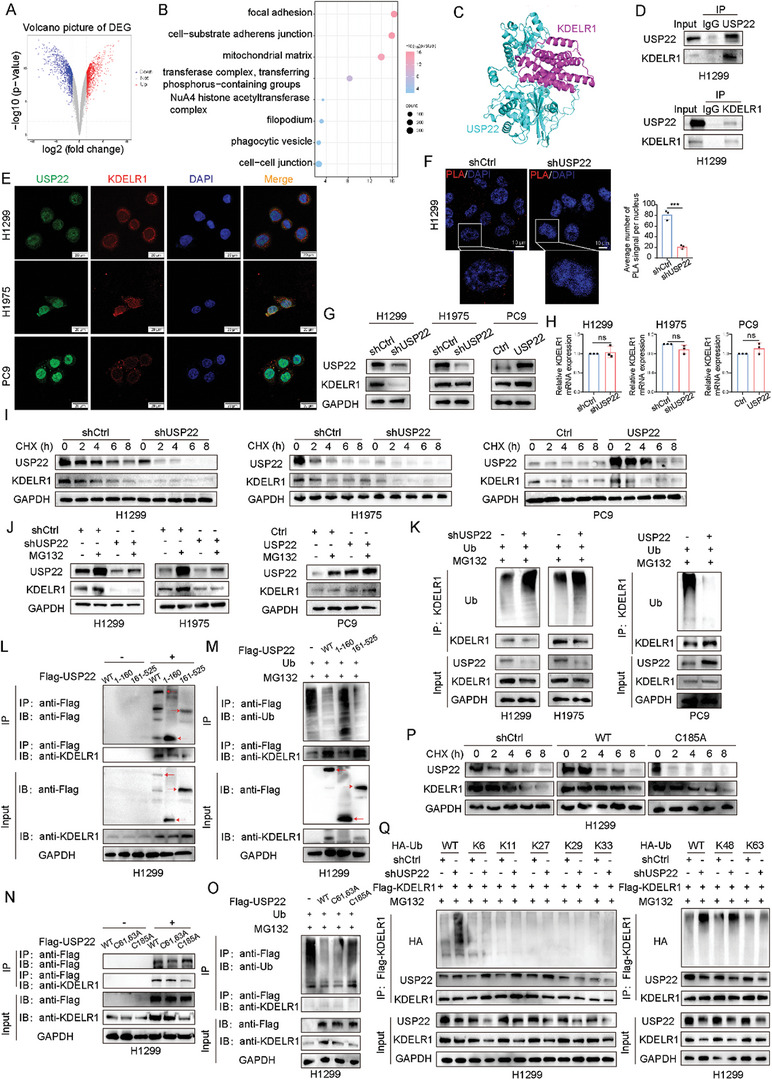
USP22 interaction with KDELR1, and USP22 effects on KDELR1 protein stabilization and ubiquitination. A) DEGs were identified after USP22 knockdown in H1975 cells. B) GO analysis of DEGs. C) Visualization of USP22‐KDELR1 binding. D) Interaction between USP22 and KDELR1 in H1299 cells verified by co‐IP assay. E) IF staining was used to examine the colocalization of USP22 and KDELR1 in LUAD cells. Scale bars, 20 µm. F) Representative images of PLA signals between USP22 and KDELR1. Scale bars, 10μm. G) Protein expression of KDELR1 in LUAD cells with USP22 knockdown and overexpression. H) mRNA expression of KDELR1 in LUAD cells with USP22 knockdown and overexpression. I) Western blotting analysis of KDELR1 treated with CHX (200 µg mL^−1^). J) Western blotting analysis of KDELR1 treated with MG132 (10 μm) for 6 h. K) Impact of USP22 knockdown and overexpression on KDELR1 ubiquitination were examined. L) Identification of the binding domains of USP22 and KDELR1. M) Effects of USP22 and its mutants on KDELR1 ubiquitination were detected via co‐IP assay. N) KDELR1 interactions with USP22 and its point mutants were examined. O) Effects of USP22 and its point mutants on KDELR1 ubiquitination were analyzed. P) Protein stability of KDELR1 was analyzed after transfection with USP22 and its point mutant plasmids. Q) KDELR1 ubiquitination assays in H1299 cells co‐transfected with HA‐Ub WT, or HA‐K6‐Ub, or HA‐K11‐Ub, or HA‐K27‐Ub, or HA‐K29‐Ub, or HA‐K33‐Ub, or HA‐K48‐Ub, or HA‐K63‐Ub plasmids after treatment with MG132 for 6h. Data are presented as mean ± SD. Statistical significance was determined by Student's *t*‐test. Not significant (ns), *p* > 0.05, ^*^ *p* < 0.05, ^**^ *p* < 0.01, ^***^ *p* < 0.001, ^****^ *p* < 0.0001.

To investigate the molecular mechanism underlying the involvement of USP22 in LUAD metastasis, we used microarray analysis to identify the molecules downstream of USP22 in H1975 cells (shCtrl and shUSP22). Analysis of DEGs in H1975 cells with depleted USP22 revealed 11560 differential genes (**Figure** **5**A). GO enrichment analysis suggested that USP22 participates in multiple cellular functions, such as focal adhesion, filopodium and cell‐substrate adherens junction (Figure 5B). Based on the National Center for Biotechnology (NCBI) database, we queried genes related to the ECM, TME, and tumor metastasis and established co‐expression modules to identify the roles of USP22 in the ECM, TME, and tumor metastasis by weighted gene co‐expression network analysis (WGCNA). The key genes of each module were identified based on the functional modules, and the key genes, KDELR1 (module 1, turquoise), NRP2 (module 2, blue), and STAT4 (module 3, brown), were obtained. Based on the association among modules with USP22 gene expression, module 1 had the strongest correlation with USP22 gene expression, and there was an up‐regulation and down‐regulation effect on LUAD cells (Figure S3A–C, Supporting Information). KDELRs is a family of receptor proteins with seven transmembrane structural domains. The KDELRs family consists of three isoforms, KDELR1, KDELR2, and KDELR3, which are mainly located in the endoplasmic reticulum and Golgi apparatus and are involved in several physiological and pathological processes, such as cellular stress response, vesicle transport, and ECM degradation.^[^ ^28^, ^29^ ^]^ KEGG analysis revealed that KDELR1 is mainly involved in regulating signaling pathways, such as the vascular endothelial growth factor (VEGF) signaling pathway, tight junctions, regulation of the actin cytoskeleton, focal adhesion, and endocytosis (Figure S3D, Supporting Information). The results from the UALCAN database showed that the protein level of KDELR1 in LUAD tissues was significantly higher than that in control tissues (Figure S3E, Supporting Information). Kaplan–Meier analysis showed a significant negative correlation between KDELR1 expression and overall survival (OS) and post progression survival (PPS) in patients with LUAD (Figure S3F,G, Supporting Information). A significant correlation between USP22 and KDELR1 in LUAD was demonstrated using GEPIA (Figure S3H, Supporting Information). We investigated the role of KDELR1 in regulating the malignant behavior of LUAD cells. We established KDELR1 knockdown and overexpression cell lines. KDELR1 was knocked down in H1299 and H1975 cells using three different small interfering RNAs (siRNAs) targeting KDELR1, and KDELR1 was overexpressed using the KDELR1 plasmid in PC9 cells (Figure S3I, Supporting Information). The results of in vitro functional experiments showed that the proliferation, migration, and invasion abilities of LUAD cells were significantly weakened after KDELR1 knockdown, whereas the proliferation, migration, and invasion abilities of LUADs were significantly enhanced after KDELR1 overexpression (Figure S3J–L, Supporting Information). Therefore, we speculated that KDELR1 may be a key gene that promotes LUAD metastasis.

We confirmed the interaction between USP22 and KDELR1 using molecular docking simulations and co‐IP assays (Figure 5C,D). IF staining confirmed the colocalization of USP22 and KDELR1 in the cytoplasm in LUAD cells (Figure 5E). The interaction between USP22 and KDELR1 was validated using a PLA assay (Figure 5F). USP22 knockdown decreased KDELR1 protein levels, whereas USP22 overexpression had the opposite effect (Figure 5G). qRT‐PCR analysis revealed that the knockdown and overexpression of USP22 had no significant effects on KDELR1 mRNA levels (Figure 5H). These results suggest that USP22 does not affect the mRNA expression level of KDELR1 and that the regulatory effect of USP22 on KDELR1 occurs at the post‐translational level. Next, we verified whether USP22 stabilizes KDELR1 expression by preventing KDELR1 degradation. USP22 knockdown markedly shortened the half‐life of KDELR1, whereas USP22 overexpression prolonged its half‐life following CHX treatment (Figure 5I). We found that the proteasome inhibitor (MG132) reversed the reduction in KDELR1 protein levels caused by USP22 knockdown (Figure 5J). We observed increased levels of KDELR1 ubiquitination after USP22 knockdown in H1299 and H1975 cell lines and decreased levels of KDELR1 ubiquitination after USP22 overexpression in PC9 cell lines (Figure 5K). These results suggested that USP22 post‐translationally regulates KDELR1 expression by preventing proteasomal degradation.

After transfecting the truncated USP22 mutants into H1299 cells, the C19 ubiquitin‐specific peptidase domain of USP22 bound strongly to KDELR1, whereas the N‐terminal zinc finger domain bound weakly to KDELR1 (Figure 5L). The C19 ubiquitin‐specific peptidase domain of USP22 was involved in KDELR1 ubiquitination (Figure 5M). Consistent with the truncated USP22 mutants, the C19 ubiquitin‐specific peptidase domain of USP22 contains a point mutation weakly bound KDELR1, whereas the N‐terminal zinc finger domain of USP22 containing a point mutation bound KDELR1 more strongly (Figure 5N). Furthermore, the point mutation in the C19 ubiquitin‐specific peptidase domain of USP22 did not affect KDELR1 ubiquitination (Figure 5O). The point mutation in the C19 ubiquitin‐specific peptidase domain of USP22 abolished its ability to increase KDELR1 protein levels following CHX treatment (Figure 5P). Using specific ubiquitin plasmids, we found that USP22 effectively cleaved the K48‐linked KDELR1 poly‐ubiquitination chain (Figure 5Q).

These results confirmed that the C19 ubiquitin‐specific peptidase domain of USP22 was the dominant mediator of the interaction between USP22 and KDELR1. USP22 catalyzed the removal of the K48‐linked polyubiquitination chain that binds to KDELR1.

### 2.5 USP22 Enhances Cell Motility and Invadopodia Formation in LUAD Cells by Stabilizing KDELR1

**Figure 6:**
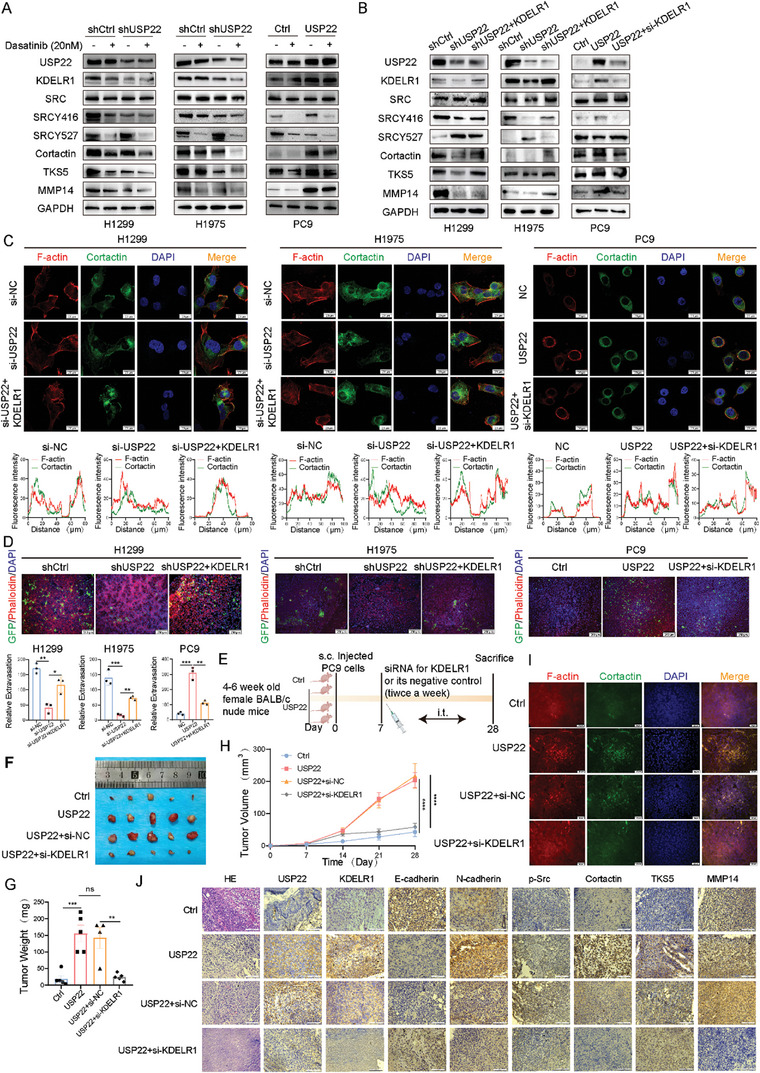
USP22 promotes the KDELR1/SRC pathway. A) Western blotting analysis of SRC cascade expression after Dasatinib treatment of USP22‐knockdown or USP22‐overexpressing cells. B) KDELR1 knockdown attenuated USP22‐mediated SRC signaling activation, whereas KDELR1 overexpression had the opposite effect. C) Invadopodia were visualized by colocalization of cortactin and F‐actin (Phalloidin). Scale bars, 20 µm. D) Trans‐endothelial migration and intravasation/extravasation by in vitro metastasis model. Scale bars, 250 µm. E) Schematic diagram of siRNA treatment. (n = 5 mice per group). F–H) In vivo analysis of tumor (F), weight (G), and proliferation curves (H) in xenograft tumors after intra‐tumoral injection of in vivo‐optimized KDELR1 inhibitor or the control twice a week. I) IF images of tissues stained with cortactin and F‐actin. Scale bars, 50 µm. J) HE and IHC staining images. Scale bars, 50 µm. Data are presented as mean ± SD. Statistical significance was determined by Student's *t*‐test and ANOVA test. Not significant (ns), *p* > 0.05, ^*^ *p* < 0.05, ^**^ *p* < 0.01, ^***^ *p* < 0.001, ^****^ *p* < 0.0001.

In our previous study, we found that USP22 is involved in the regulation of filopodium (Figure 5B) and that KDELR1 is associated with the regulation of the actin cytoskeleton (Figure S3D, Supporting Information). Therefore, we hypothesized that the USP22‐KDELR1 axis regulates the invadopodia process to drive LUAD metastasis. The SRC signaling pathway is closely related to invadopodia formation, maturation, and function.^[^ ^30^ ^]^ Therefore, we investigated the effects of USP22 and KDELR1 on the SRC signaling pathway. Next, we used the SRC inhibitor, Dasatinib (20 nM), to examine whether USP22 could activate SRC signaling in LUAD cells. Western blotting revealed that USP22 activated SRC, which was inhibited by Dasatinib (**Figure** **6**A). The SRC activity is regulated by tyrosine phosphorylation at two sites. The phosphorylation of Tyr416 upregulates enzyme activity, whereas the phosphorylation of Tyr527 reduces enzyme activity.^[^ ^31^ ^]^ Moreover, we observed that the levels of invadopodia markers (cortactin and TKS5) and MMP14 were reduced after Dasatinib treatment. To verify whether USP22 activates the SRC pathway by stabilizing KDELR1 expression, we overexpressed KDELR1 in H1299 and H1975 cells with USP22 knockdown and depleted KDELR1 in PC9 cells with USP22 overexpression. Western blotting analysis showed that KDELR1 knockdown by siRNA transfection after USP22 overexpression abrogated USP22‐induced SRC pathway activation and the expression of invadopodia markers and MMP14 in PC9 cells. Overexpression of KDELR1 had the opposite effect on H1299 and H1975 cells with USP22 knockdown (Figure 6B). Given that these findings suggest that USP22 regulates KDELR1, we investigated the effect of KDELR1 on USP22‐induced LUAD carcinogenesis. We then performed transwell and wound healing assays. These results indicate that KDELR1 is necessary for USP22‐mediated invasion and migration of LUADs (Figure S4A,B, Supporting Information). KDELR1 enhanced the USP22‐mediated invadopodia formation and promoted the ability of tumor cells to penetrate the basement membrane in the in vitro metastasis model (Figure 6C,D).

To better understand the role of KDELR1 in tumorigenesis in vivo, we established USP22 control and overexpression tumor models and intratumoral injection of siRNA for KDELR1 (5 nmol/20 g/mice) (Figure 6E). KDELR1 depletion using in vivo‐optimized RNAi significantly weakened tumor growth (Figure 6F–H). Silencing of KDELR1 significantly attenuated invadopodia formation compared with that in the control group (Figure 6I). IHC staining showed that silencing KDELR1 reduced SRC activation and the expression of invadopodia markers and matrix metalloenzymes (Figure 6J).

In conclusion, USP22 activates the SRC signaling pathway by stabilizing KDELR1, which promotes invadopodia formation by LUAD cells.

### 2.6 Identification of a USP22‐Specific Inhibitor

We examined the dose response of Usp22i‐S02 in LUAD cells and found that Usp22i‐S02 did not inhibit USP22 expression and had little effect on cell viability (Figure S5A,B, Supporting Information).^[^ ^32^ ^]^ Next, based on natural drug active substances used in tumor therapy, we selected berberine (BBR), an isoquinoline alkaloid found in Coptis chinensis structurally similar to Usp22i‐S02 for further study. Previous studies have reported that BBR has significant preventive and curative effects on a variety of common tumors, such as breast,^[^ ^33^ ^]^ lung,^[^ ^34^ ^]^ liver,^[^ ^35^ ^]^ stomach,^[^ ^36^ ^]^ and colon cancers.^[^ ^37^ ^]^ Therefore, we further explored the cancer‐inhibitory effects of BBR analogs on LUAD cells. We examined the effects of the 13 compounds on USP22 expression using western blotting (Table S1, Supporting Information). The results showed that Epiberberine, Groenlandicine, and 13‐methylberberine (13‐MB) inhibit the expression of USP22 (Figure S5C, Supporting Information). We further selected Epiberberine, Groenlandicine, and 13‐MB, which had significant inhibitory effects on USP22 expression, and CCK‐8 assays were used to determine their effects on the proliferation of H1299 cells. The inhibitory effect of 13‐MB on the proliferation of H1299 cells was most observed after 24 and 48 h of administration compared with that of the control group and showed a significant concentration dependence (Figure S5D, Supporting Information). The UBP8 structure [Protein Data Bank (PDB) code 3MHS] was selected as the template protein to model USP22 using the Swiss model (Figure S5E, Supporting Information). USP22 and 13‐MB structures are shown in Figure S5F (Supporting Information). Docking analysis was performed using the AutoDock software to elucidate the interaction between USP22 and 13‐MB. The root mean square deviation (RMSD) trajectory showed stable binding of 13‐MB to the USP22 catalytic domain pocket (Figure S5G–I, Supporting Information). Amino acid residues are involved in 13‐MB interactions: ILE268 produces a *π*‐sigma interaction with the compound, and TRP325 produces a *π–π* interaction with the compound (Figure S5G, Supporting Information).

**Figure 7:**
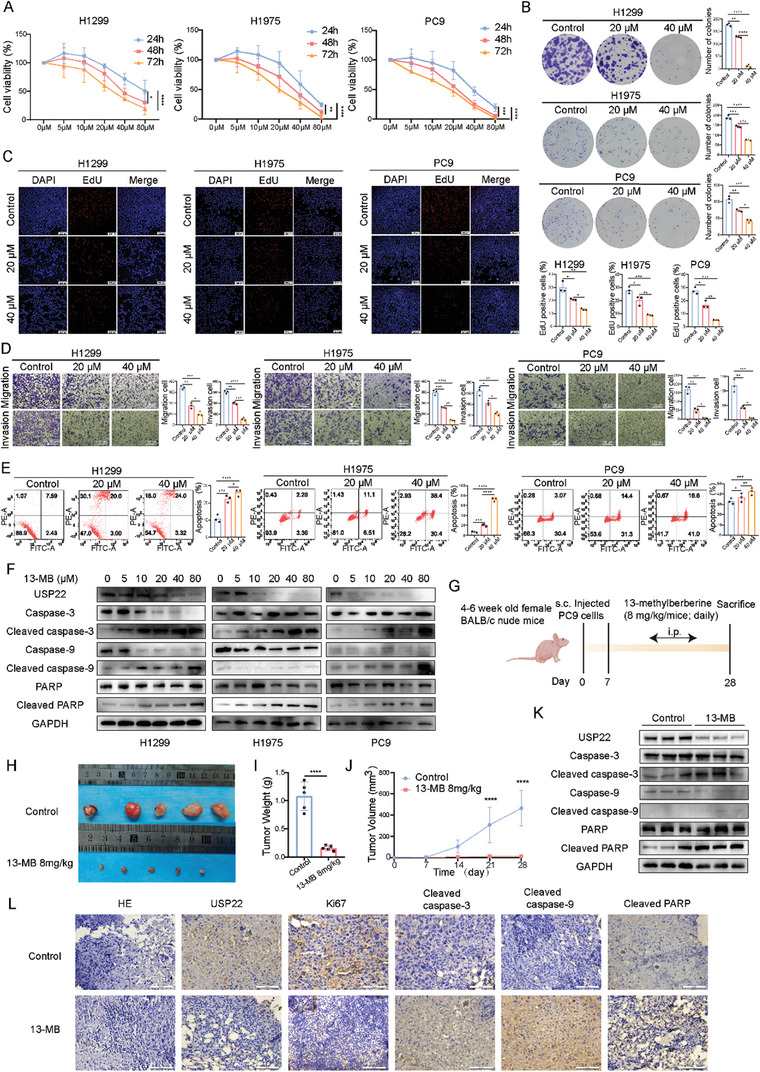
Antitumor effect of 13‐MB on LUAD in vitro and in vivo. A) Effect of different concentrations of 13‐MB after 24, 48, and 72 h on the survival fraction of LUAD cells. B,C) Effect of 13‐MB (20 and 40 µm) on the proliferative capacity of LUAD cells as detected by clone formation (B) and EdU assays (C). D) Results of transwell assay for detecting the migratory and invasive abilities of cells treated with 13‐MB. E) Apoptosis of LUAD cells treated with different concentrations of 13‐MB for 24 h, which were stained with fluorescein isothiocyanate (FITC)‐conjugated Annexin V antibody and propidium iodide (PI) before flow cytometry. F) LUAD cells were treated with different concentrations of 13‐MB for 24 h. Western blotting analysis of the protein expression levels of USP22, caspase‐3, cleaved caspase‐3, caspase‐9, cleaved caspase‐9, PARP, and cleaved PARP. G) Schematic diagram of the effects of 13‐MB in vivo. (n = 5 mice per group). H–J) Tumor images (H), growth weights (I), and proliferation curves (J). K) Western blotting analysis of the expression of USP22 and apoptosis indicators in tumor tissues. L) Tumor tissues were stained for USP22, Ki67, cleaved caspase‐3, cleaved caspase‐9, and cleaved PARP using HE and IHC staining. Scale bars, 25 µm. Data are presented as mean ± SD. Statistical significance was determined by Student's *t*‐test and ANOVA test. ^*^ *p* < 0.05, ^**^ *p* < 0.01, ^***^ *p* < 0.001, ^****^ *p* < 0.0001.

The effect of 13‐MB on tumor cell proliferation was further investigated, revealing significant concentration‐ and time‐dependent inhibition of H1299, H1975, and PC9 cells by 13‐MB (**Figure** **7**A). Using colony formation and EdU assays, we found that 13‐MB significantly inhibited the proliferation of LUAD cells (Figure 7B,C). The results of the transwell assay of LUAD cells with and without the addition of 13‐MB showed that the migration and invasion abilities of the cells were significantly limited after the addition of 13‐MB, and the higher the concentration of 13‐MB, the more the inhibitory effect (Figure 7D). Flow cytometry revealed that the apoptotic rate of the cells was significantly higher than that of the control after treatment with 13‐MB (Figure 7E). Western blotting results showed that apoptotic proteins were more obvious at higher drug concentrations (Figure 7F). These findings suggested that 13‐MB may limit LUAD cell carcinogenesis in vitro in several ways. To further validate the effects of 13‐MB on LUAD cells, we established a subcutaneous LUAD xenograft tumor model (Figure 7G). After tumor formation, one group of mice was injected intraperitoneally with saline, and another group was injected intraperitoneally with 13‐MB. The tumor tissues from the mice injected with 13‐MB were significantly reduced compared with those in the control group (Figure 7H‐J). We performed western blotting experiments to further confirm the protein levels in both tumor groups. Caspase‐3, caspase‐9, and PARP levels in tumor tissues did not change significantly after 13‐MB treatment; however, cleaved caspase‐3, cleaved caspase‐9, and cleaved PARP expression were significantly enhanced, and USP22 expression was significantly inhibited (Figure 7K). The IHC staining revealed decreased USP22 and Ki67 expression and increased cleaved caspase‐3, cleaved caspase‐9, and cleaved PARP expression after 13‐MB treatment (Figure 7L).

These results suggest that 13‐MB acts as an inhibitor of USP22 to inhibit the progression of LUAD.

## 3 Discussion

EVs play important roles in cellular communication and many biological processes. Several studies have shown that EVs are involved in various oncogenic processes, including metastasis, angiogenesis, immune evasion, and chemoresistance. EVs biogenesis involves four key steps: cargo sorting, MVB formation and maturation, MVB transport, and MVB fusion with the plasma membrane.^[^ ^38^ ^]^ Small Rab GTPases (including Rab11, Rab27, and Rab35) play a key role in intracellular vesicle trafficking by affecting the transport or docking of MVBs to the plasma membrane.^[^ ^39^ ^]^ The SNARE complex consists of three to four SNARE proteins located on vesicles and target membranes, and multiple SNAREs with different compositions mediate MVB‐plasma membrane fusion in different cells.^[^ ^40^ ^]^ Considerable progress has been made concerning the important role of EVs in cancer progression; however, there are relatively few studies on the mechanism of EV secretion. Cancer regulates EV biogenesis and promotes the release of tumor‐promoting EVs through various strategies. Therefore, understanding the mechanisms underlying EV secretion is a promising therapeutic strategy.

Our previous study showed that USP22 promoted LUAD metastasis both in vivo and in vitro. The expression level of USP22 protein in the tumor tissues of patients with LUAD was significantly higher than that in adjacent normal tissues, and patients with high USP22 expression had a worse prognosis than those with low USP22 expression. We found that USP22 was localized to late endosomes, known as MVBs.^[^ ^17^ ^]^ Therefore, we speculated that USP22 plays a role in the later stages of EV biogenesis, probably during MVB transport or fusion with the plasma membrane. In the present study, USP22 promoted the secretion of EVs in LUAD cells and xenograft models. USP22 Knockdown or overexpression decreased and increased the secretion of EVs, respectively, in LUAD cells (Figure 1). MS analysis showed that USP22 interacted with MYO1B in H1299 cells. Myosin plays a key role in cancer development and progression. As regulators of vesicular transport, myosin can control interactions within cells or between cells and the environment.^[^ ^22^ ^]^ MYO1B is involved in the morphological organization of early sorting MVBs and play a role in the transfer of cargo proteins to the internal vesicles of endosomes.^[^ ^41^ ^]^ In addition, myosin acts as a molecular motor, and the coupling between myosin movement and actin track assembly is indispensable during vesicle long‐distance transport.^[^ ^42^ ^]^ MYO1B is stabilized by its interaction with USP22, thereby preventing ubiquitination and degradation via the ubiquitin‐proteasome pathway (Figure 2). Summarily, we demonstrated that USP22 controls MVB transport to promote the release of EVs by inhibiting proteasomal degradation of MYO1B.

USP22, a deubiquitinating enzyme, is reportedly associated with several aggressive phenotypes in many tumors. USP22 can affect many oncogenic signaling cascades, such as tumor metastasis,^[^ ^43^ ^]^ immune evasion,^[^ ^44^ ^]^ and cell growth.^[^ ^12^ ^]^ Although many studies have investigated the function of USP22 as an intracellular protein, we demonstrated for the first time that USP22 can be transported through EVs and promote tumor progression by promoting EV secretion. Next, we isolated tumor cell‐derived EVs by differential ultracentrifugation and proteomic sequencing. KEGG and GO enrichment analysis revealed that USP22‐mediated tumor cell‐derived EVs are mainly involved in signaling pathways, such as cell motility and migration. We demonstrated that USP22 in tumor cell‐derived EVs promoted tumor metastasis and invadopodia formation and enhanced tumor cell motility in vitro (Figure 3). The in vivo experiments showed that PC9 cells with USP22 overexpression‐derived EVs promoted tumor growth and malignant phenotypic transformation (Figure 4).

To further explore the molecular mechanisms by which USP22‐mediated tumor cell‐derived EVs promote tumor metastasis in LUADs, we identified KDELR1 as a downstream effector of USP22 using gene chip and bioinformatics analyses (Figure 5). Previous studies have suggested that the main function of KDELR is the retrograde transport of endoplasmic reticulum‐resident proteins from the Golgi complex to the endoplasmic reticulum.^[^ ^29^ ^]^ Currently, the molecular mechanisms of KDELRs in tumors are only partially understood and include membrane trafficking,^[^ ^45^ ^]^ cytoskeletal reorganization,^[^ ^46^ ^]^ and activation of the mTORC1 pathway.^[^ ^47^ ^]^ KDELR1 and KDELR2 stimulate invadopodia assembly and ECM degradation, and these effects are closely linked to the activation of the SRC signaling pathway.^[^ ^46^, ^48^ ^]^ However, the role of KDELR1 in the progression of LUAD remains unclear. We found that KDELR1 promotes the proliferation, invasion, and migration of tumor cells and that high expression of KDELR1 was associated with poor prognosis in patients with LUADs. Our study showed that USP22 and KDELR1 are co‐localized in LUAD cells. High levels of USP22 activated the SRC cascade, which was inhibited by Dasatinib, whereas there was no difference in the USP22 and KDELR1 levels. In addition, the expression of the SRC signaling pathway decreased after KDELR1 knockdown. KDELR1 knockdown reversed the role of USP22 in promoting invadopodia formation and tumor metastasis (Figure 6). These results suggest that USP22 activates the SRC signaling pathway through KDELR1 interaction and that the SRC pathway promotes invadopodia formation and metastasis.

BBR is an alkaloid isolated from Rhizoma coptidis, and many studies have demonstrated the high activity of BBR and its derivatives against tumors. BBR analogs have a stronger effect on cell proliferation and apoptosis than BBR.^[^ ^49^ ^]^ In this study, we evaluated the effects of 13 BBR analogs on LUAD cells. These 13 compounds were structurally similar to BBR. We found that 13‐MB significantly inhibited USP22 expression, cell proliferation, and migration and promoted apoptosis of LUAD cells. Finally, we examined the in vivo antitumor effects of 13‐MB. The 13‐MB treatment had a significant inhibitory effect on tumor growth. In addition, the IHC staing showed that USP22 and Ki67 levels decreased, whereas the levels of cleaved caspase‐3, cleaved caspase‐9, and cleaved PARP increased in 13‐MB‐treated tumor xenograft mice, indicating that 13‐MB triggered significant apoptosis (Figure 7). This study demonstrates that 13‐MB inhibits USP22 expression and thus suppresses tumor progression, supporting its potential application as a small‐molecule inhibitor of USP22.

**Figure 8:**
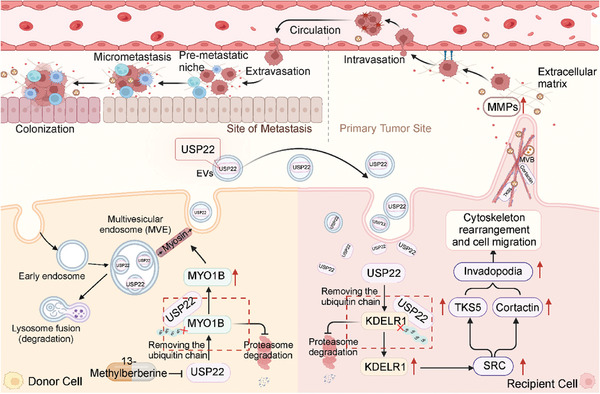
The mechanisms of USP22 mediates tumor cell‐derived EV secretion to promote tumor motility.

These findings confirm that USP22 regulates MVB transport and fusion with the plasma membrane, thus participating in EV secretion. USP22‐mediated EV secretion contributes to invadopodia formation in LUAD cells, which in turn promotes tumor cell invasion (**Figure** **8**). Notably, invadopodia are specific and critical docking and secretion sites for MVBs, and the secretion of MVBs plays a role in the invadopodia formation, thus forming a positive feedback loop to promote tumor metastasis.

## 4 Experimental Section

### Cell Culture and Transfection

Human lung cancer cell lines were maintained at our laboratory (Cancer Research Institute, Harbin Medical University). NCI‐H1299 and NCI‐H1975 cells were cultured in Roswell Park Memorial Institute‐1640 medium (Gibco), and PC9 cells were cultured in Dulbecco's modified eagle medium (Gibco), and all of them were supplemented with 10% fetal bovine serum (FBS) (Cell‐box, #AUS‐01S‐02) and incubated at 37 °C and 5% CO_2_. The cells were verified using short tandem repeat (STR) sequence analysis. All the experiments were performed using mycoplasma‐free cells. Lentivirus vectors with UPS22 overexpression and USP22 shRNA were purchased from GeneChem. Lentiviruses with USP22 knockdown and overexpression were transfected with cells containing 5 µg mL^−1^ polybrene. Cells were stabilized by screening with 2 µg mL^−1^ puromycin. USP22 and KDELR1 siRNAs were purchased from RiboBio. Plasmids with USP22 and KDELR1 overexpression were purchased from SinoBiological. All siRNAs and plasmids were transfected with jetPRIME according to the manufacturer's instructions. The sequences targeted by the siRNAs were as follows: siUSP22#1 (5′‐CACGGACAGTCTCAACAAT‐3′), siUSP22#2 (5′‐CTGCAAAGGTGATGACAAT‐3′); siKDELR1#1 (5′‐CCAACTACATCTCACTCTA‐3′), siKDELR1#2 (5′‐CTACCTCTATATCACCAAA‐3′), siKDELR1#3 (5′‐GGTGTTCACTGCCCGATAT‐3′).

### EVs Isolation, Characterization, and Fluorescent Labelling

EVs were isolated by differential ultracentrifugation and characterized as previously described.^[^ ^50^ ^]^

### RNA Isolation and qRT‐PCR

Total RNA was extracted from the cultured cells using TRIzol reagent (Roche). RNA (1000 ng) from each sample was reverse transcribed into cDNA using a Transcriptor First Strand cDNA Synthesis Kit (Roche). The target genes were quantified using a Light Cycler 480 II (Roche). GAPDH was used as the internal reference. The primer sequences are listed in Table S2 (Supporting Information).

### Immunofluorescence (IF) Staining

The cells were fixed with 4% paraformaldehyde, permeabilized with 0.1% Triton X‐100 for 10 min, and blocked with 5% bovine serum albumin (BSA) for 30 min. The cells were incubated with primary antibodies at 4 °C overnight. Secondary antibodies were added in the dark at room temperature for 1 h, and the cells were stained with 4, 6‐diamino‐2‐phenylindole (DAPI). Finally, the stained cells were observed and photographed using a confocal microscope (Zeiss).

### Immunohistochemical (IHC) and Hematoxylin and Eosin (HE) Staining

Tissues were fixed in 4% paraformaldehyde, embedded in paraffin, sectioned, and stained with primary antibodies. Tissue sections were subjected to antigenic repair using sodium citrate or ethylenediaminetetraacetic acid (EDTA). Non‐specific binding sites on the tissue sections were blocked with 4% BSA. Tissues were incubated with specific primary antibodies overnight at 4 °C. The next day, horseradish peroxidase (HRP)‐labeled secondary antibodies were added to the tissue sections. The tissue sections were stained using 3,3′‐diaminobenzidine (DAB). The tissue sections were stained with hematoxylin. Tissue sections were stained with HE (G1120, Solarbio) for morphological observations. The stained tissue sections were imaged using a microscope.

### Cell Proliferation Assay

For the CCK‐8 assay, the cells were digested in a cell suspension of a certain density and placed in 96‐well plates at 3 000–5 000 cells per well. After a certain period of applying the interventions, the culture medium was discarded. CCK‐8 reagent (Seven, Beijing, China) and the culture medium (1:9) were then added (100 µL per well). After incubation at 37 °C for 2 h, the absorbance of the samples was measured at 450 nm using a microplate reader. The presence of 5‐ethynyl‐2‐deoxyuridine (EdU) was detected in the cells using an EdU Kit (RiboBio) according to the manufacturer's instructions. The cells were seeded in 96‐well plates at a density of 10 000 cells per well. For the colony formation assay, cells were seeded in six‐well plates at a density of 600–1000 cells per well. Thereafter, the cells were incubated at 37 °C in 5% CO_2_ for 2 weeks. Subsequently, colonies were fixed with paraformaldehyde, stained with crystal violet, and counted.

### Transwell and Wound Healing Assays

For transwell assay, the cells (3–5 × 10^4^ cells) were suspended in 200 µL of serum‐free medium and seeded in the upper chamber with or without Matrigel (BD Biosciences). The lower chamber was filled with 600 µL of 10% FBS. After 24–48 h (migration: 24 h; invasion: 48 h), cells at the bottom were fixed with 4% paraformaldehyde for 20 min and stained with crystal violet for 20 min. Five randomly selected fields of view in each chamber were photographed, and the cells were counted. For wound healing assays, cells were incubated in a six‐well plate until 95% of the bottom of the plate was covered. Artificial wounds were scratched on confluent cells using a 10 µL pipette tip. Wound healing images were captured at 0, 24, and 48 h.

### Tube Formation

Tube formation assay was performed previously as described.^[^ ^50^ ^]^

### Western Blotting Analysis

Western blotting was performed as previously described.^[^ ^17^ ^]^ Briefly, cells were lysed on ice in RIPA buffer, and protein concentrations were determined. Equal amounts of proteins were subjected to electrophoresis on SDS‐PAGE gels, followed by immunoblot assays using the antibodies listed in Table S3 (Supporting Information).

### Co‐Immunoprecipitation (Co‐IP) Assay and Mass Spectrometry (MS) Analysis

Co‐IP assays were performed to identify interactions between different proteins. Complexes were precipitated using protein A/G magnetic beads (MedChemExpress), followed by western blotting. H1299 cell lysates were immunoprecipitated using anti‐USP22 or anti‐IgG antibodies. The protein complexes eluted from the beads were subjected to MS analysis. Based on the identified amino acid sequences, USP22‐interacting client proteins were identified using UniProt.

### Ubiquitination Assay

Ubiquitinated plasmid was used to overexpress ubiquitin, and the cells were treated with the proteasome inhibitor MG132 (10 µm) for 6 h and lysed. Co‐IP was performed using primary antibodies. Ubiquitination levels were examined via western blotting using an anti‐ubiquitin antibody.

### Proximity Ligation Assay (PLA)

The interactions among USP22, and MYO1B, USP22, and KDELR1 were assessed using a Duolink in situ PLA kit (Sigma–Aldrich, DUO92101) according to the manufacturer's instructions. The PLA Signals from the cells were imaged using a confocal microscope (Zeiss).

### Flow Cytometry for Cell Apoptosis Assays

H1299, H1975, and PC9 cells were seeded into 25 cm^2^ flasks at a density of 5 × 10^5^ cells per bottle. The cells were then treated with the vehicle, 20 or 40 µm 13‐MB for 24 h. Apoptosis was detected using the Annexin V‐PI Apoptosis Assay Kit (Seven, Beijing, China). Cells were first digested with trypsin without EDTA, harvested, resuspended in binding buffer, and stained with Annexin V‐FITC and PI for 15 min. Apoptotic cells were analyzed using flow cytometry (BD FACSCalibur).

### In Vitro Metastasis Model

HUVECs (2 × 10^5^ cells) seeded in Matrigel invasion chambers (pore size, 8 µm; BD Biosciences). Medium containing 10% FBS was added to the lower chamber. After 24 h, 3–5 × 10^4^ cancer cells stably expressing GFP were resuspended in a serum‐free medium and added to the upper chamber. The 24‐well plates were incubated in a CO_2_ incubator for 3–7 days. The upper chamber was removed, fixed with 4% paraformaldehyde for 20 min, permeabilized with a 0.1% Triton X‐100 for 10 min, and stained with phalloidin (red) and DAPI. Trans‐endothelial‐migrated LUAD cells that passed through the HUVECs were imaged and counted using a fluorescence microscope (Leica).

### Establishment of Tumor in Nude Mice

Animal experiments were performed in accordance with the guidelines approved by the Ethics Committee of the Institutional Animal Care and Use Committee of Harbin Medical University. BALB/c nude mice (4–5 weeks) were obtained from the Vital River Laboratory. H1299 cells (5 × 10^6^ cells in 100 µL of PBS) were stably transfected with an empty control vector, and shUSP22 cells were subcutaneously injected into the backs of mice. The tumor size was measured using a vernier caliper every 7 days. The tumor volume was calculated using the formula: volume  =  length × width^2^/2. All mice were sacrificed 4 weeks after the subcutaneous injection, and the tumors were collected. A portion of the tumor tissue was fixed in a 4% paraformaldehyde solution for HE, IF, and IHC staining analyses. Another portion of the tissue was frozen in dry ice for proteomic sequencing, and the rest was stored at −80 °C for western blotting analyses.

PC9 cells (5 × 10^6^ cells in 100 µL of PBS) were subcutaneously injected into the mice, and after 1 week, the mice were randomly divided into three groups. EVs (1 × 10^9^ particles) extracted from control and PC9 cells with USP22 overexpression were intratumorally injected twice a week. The subcutaneous tumor volume was measured weekly. After 5 weeks of subcutaneous tumor formation, the nude mice were sacrificed. The volume and weight of the subcutaneous tumors were measured, and western blotting, HE, IHC, and IF analyses were conducted.

To assess the in vivo function of KDELR1, the mice were injected subcutaneously with control or PC9 cells with UPS22 overexpression (5 × 10^6^ cells in 100 µL of PBS). The mice were injected intratumorally with cholesterol‐modified KDELR1 siRNA or control siRNA (Ribobio, 5 nmol/20 g/mice) dissolved in diluted water twice a week for 3 weeks. Tumor tissues were dissected for HE, IHC, and IF staining after sacrificing the mice.

The mice were subcutaneously injected with PC9 cells (5 × 10^6^ cells in 100 µL of PBS), and after 1 week, the mice were randomized into vehicle control and treatment groups. 13‐MB (8 mg/kg body weight) was dissolved in 100 µL of vehicle solution (saline containing 0.5% dimethyl sulfoxide (DMSO)) and administered intraperitoneally to the mice daily for 21 days. The mice were sacrificed after the last injection, and tumors were collected for IHC and western blot analysis.

### Molecular Docking

AlphaFold‐predicted structures of USP22 and KDELR1 were downloaded from the UniProt database (https://www.uniprot.org/). The crystal structure of MYO1B (PDB: 4L79) was downloaded from the RCSB Protein Data Bank (PDB; https://www.rcsb.org/). Protein‐protein docking was performed using the GRAMM online server (https://gramm.compbio.ku.edu/), and the interaction force between two proteins was analyzed using the PDBePISA (https://www.ebi.ac.uk/msd‐srv/prot_int/) website after obtaining the top ten docked poses. The docking simulations of 13‐MB and USP22 were predicted using the AutoDock Vina software. The binding stability of the complexes was determined by analyzing the RMSD.

### Bioinformatics Analysis

The Kaplan–Meier Plotter (http://www.kmplot.com), UALCAN (http://ualcan.path.uab.edu), and GEPIA (http://gepia2.cancer‐pku.cn) were used for bioinformatics analysis. Survival data for patients with lung adenocarcinoma were downloaded from the Kaplan–Meier Plotter and analyzed using the Kaplan–Meier analysis and log‐rank test. The LUAD dataset was used to analyze the expression levels of KDELR1 using UALCAN. The correlation between USP22 and KDELR1 expression in lung adenocarcinoma was determined using GEPIA.

### Statistical Analyses

All experiments were performed in triplicates. Comparisons within two treatment groups were compared using the Student's unpaired two‐tailed *t*‐test. Multiple group comparisons were performed using a two‐way analysis of variance (one‐way ANOVA) using GraphPad Prism. Data were expressed as means ± SD, with *p* < 0.05 considered statistically significant. Not significant (ns), ^*^ *p* < 0.05, ^**^ *p* < 0.01, ^***^ *p* < 0.001, ^****^ *p* < 0.0001.

## Conflict of Interest

The authors declare no conflict of interest.

## Author Contributions

F.Z., Y.S., and H.W. contributed equally to this work. F.Z., Y.S., H.W., and J.H. designed the research. F.Z., Y.S., and H.W. carried out experiments. W.L., X.L., and Y.W. provided support with data analysis. Q.W. and J.H. wrote the paper and critically reviewed the manuscript. All authors read and approved the final manuscript.

## Supporting information

Supporting Information

Supporting Information

## Data Availability

The data that support the findings of this study are available in the supplementary material of this article.
