# Supplementary material for: Ubiquitin‐Specific Protease 22 Plays a Key Role in Increasing Extracellular Vesicle Secretion and Regulating Cell Motility of Lung Adenocarcinoma
Source: Adv Sci (Weinh). 2024 Aug 5;11(38):2405731. doi: 10.1002/advs.202405731 (PMC11481270; doi:10.1002/advs.202405731)
Supplement: Supplementary file 1 — Supporting Information [file ADVS-11-2405731-s001.docx]

Supplementary Materials for

Ubiquitin-specific protease 22 plays a key role in increasing extracellular vesicle secretion and regulating cell motility of lung adenocarcinoma

Fang Zhen et al.

Corresponding author: Jing Hu, [hujing@ems.hrbmu.edu.cn](mailto:hujing@ems.hrbmu.edu.cn)


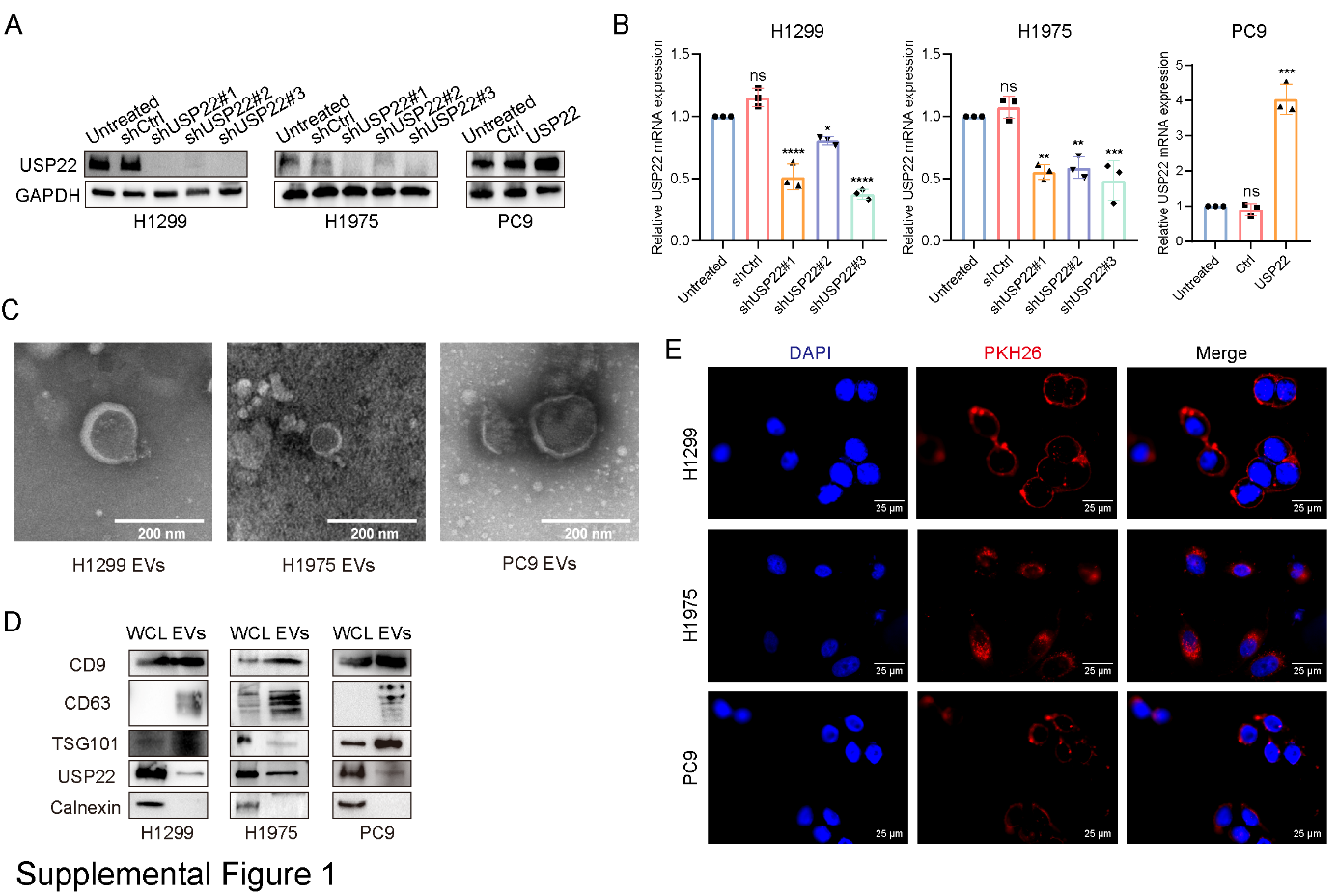


**Figure S1. Identification of tumor cell-derived EVs in LUAD cell lines.** (**A**) Western blotting validation of USP22 knockdown and overexpression efficiency at the protein level in LUAD cell lines. (**B**) qRT-PCR validation of USP22 knockdown and overexpression efficiency at the mRNA level. (**C, D**) Characterization of EVs using TEM (**C**) and western blotting (**D**) to detect EV-derived markers (CD9, CD63, and TSG101) and USP22 in whole-cell lysates and EVs. (**E**) Confocal microscopy images of PKH26-labeled EVs endocytosis in LUAD cells. Scale bars, 25μm. Data are presented as mean ± SD. Statistical significance was determined by ANOVA test. Not significant (ns), *p* > 0.05, **p* < 0.05, ***p* < 0.01, ****p* < 0.001, *****p* < 0.0001.


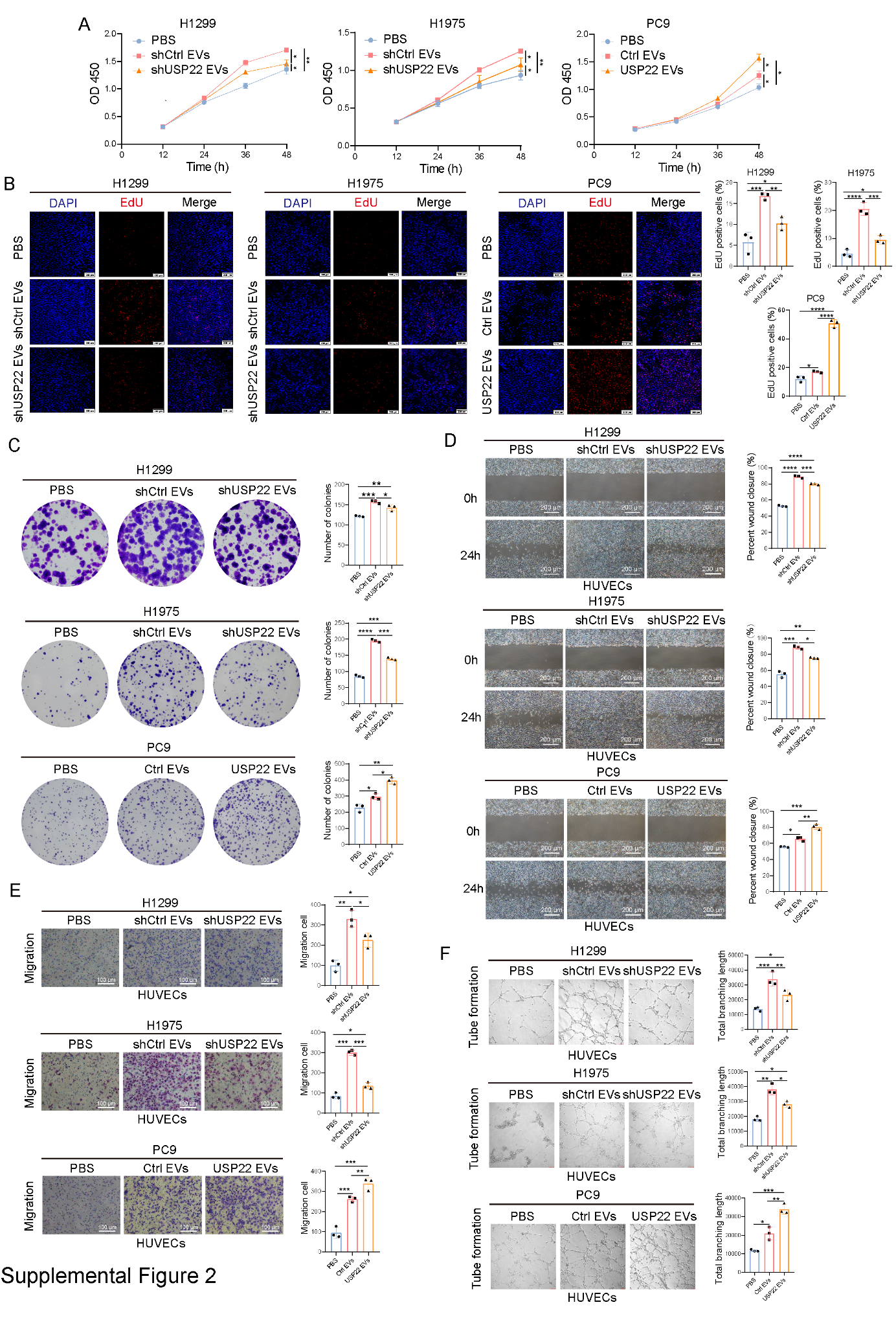


**Figure S2. EVs-derived USP22 promotes malignant properties of LUAD.** (**A, B**) Proliferative ability of LUAD cells treated with EVs was examined using CCK-8 (**A**) and EdU incorporation assays (**B**). Scale bars, 200 μm. (**C**) Colony formation ability of LUAD cells treated with EVs was detected using a colony formation assay. The cell migration ability of HUVECs treated with EVs was detected by wound healing (**D**) and transwell migration assays (**E**). (**F**) Angiogenic ability of HUVECs treated with EVs was detected using tube formation assays. Scale bars, 100 μm. Data are presented as mean ± SD. Statistical significance was determined by ANOVA test. **p* < 0.05, ***p* < 0.01, ****p* < 0.001, *****p* < 0.0001.


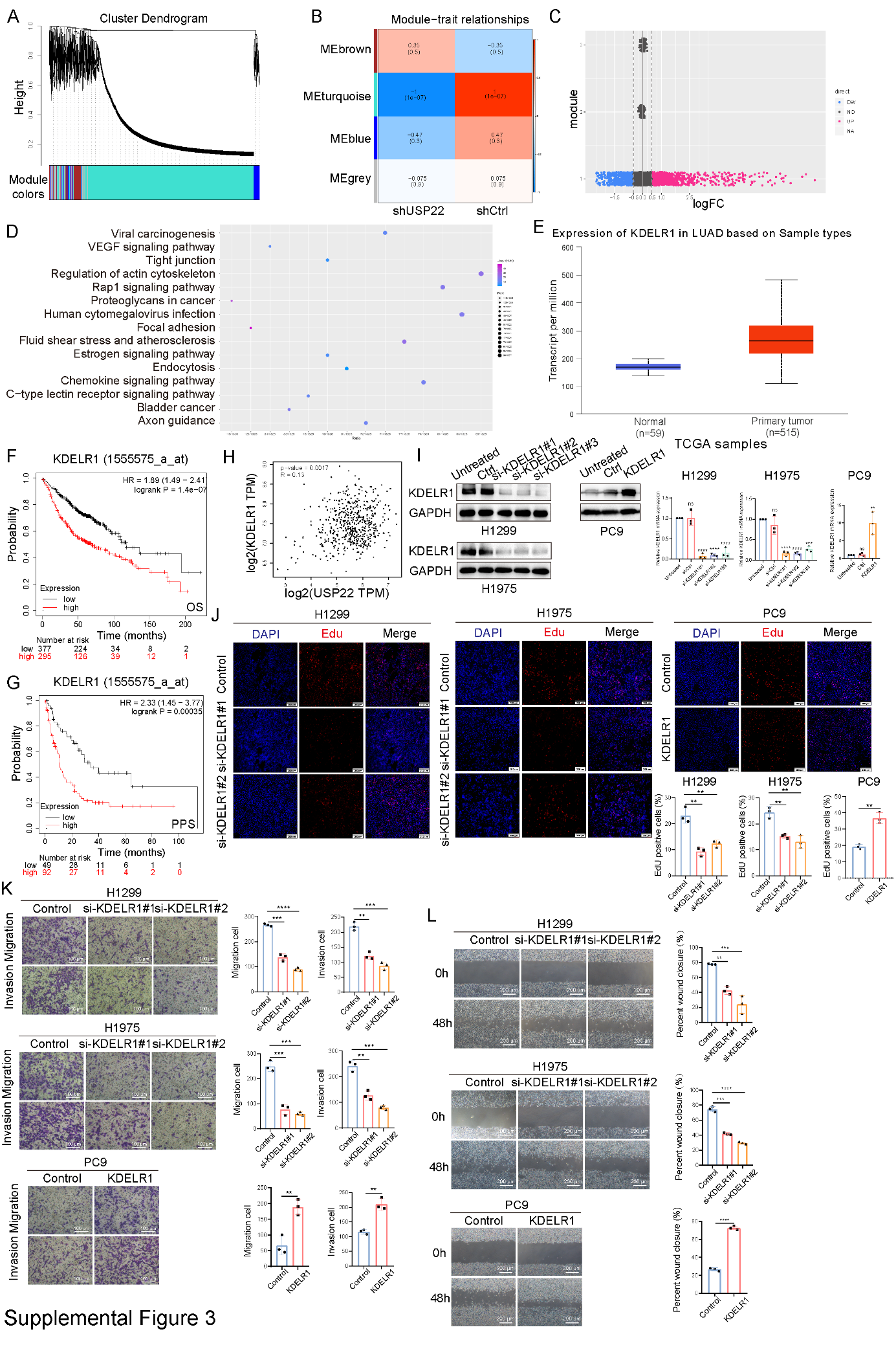


**Figure S3. KDELR1 supports the tumorigenic properties of LUAD cells.** (**A**) Clustering dendrogram of the involved genes. (**B**) Heatmap of the correlation between MEs and USP22 expression. (**C**) Module 1: turquoise, module 2: blue, module 3: brown; module 1 had the strongest correlation with USP22 gene expression. (**D**) KEGG pathway analysis of KDELR1. (**E**) Analysis of KDELR1 expression using the UALCAN database. (**F, G**) Kaplan–Meier survival curves to evaluate the OS and PPS of KDELR1 expression in patients with LUAD patients. (**H**) Correlation analysis of USP22 with KDELR1. Data were downloaded from the GEPIA2. (**I**) Knockdown and overexpression efficiencies of KDELR1 at protein and mRNA levels in LUAD cells. (**J**) EdU incorporation assay was used to determine the effect of KDELR1 on LUAD cell proliferation. Scale bars, 200 μm. (**K**) Transwell assays were performed to examine the effects of KDELR1 on the migration and invasion of LUAD cells. Scale bars, 100 μm. (**L**) Wound healing assays revealed the effect of KDELR1 on cell migration. Scale bars, 200 μm. Data are presented as mean ± SD. Statistical significance was determined by Student’s t test and ANOVA test. Not significant (ns), *p* > 0.05, **p* < 0.05, ***p* < 0.01, ****p* < 0.001, *****p* < 0.0001.

**
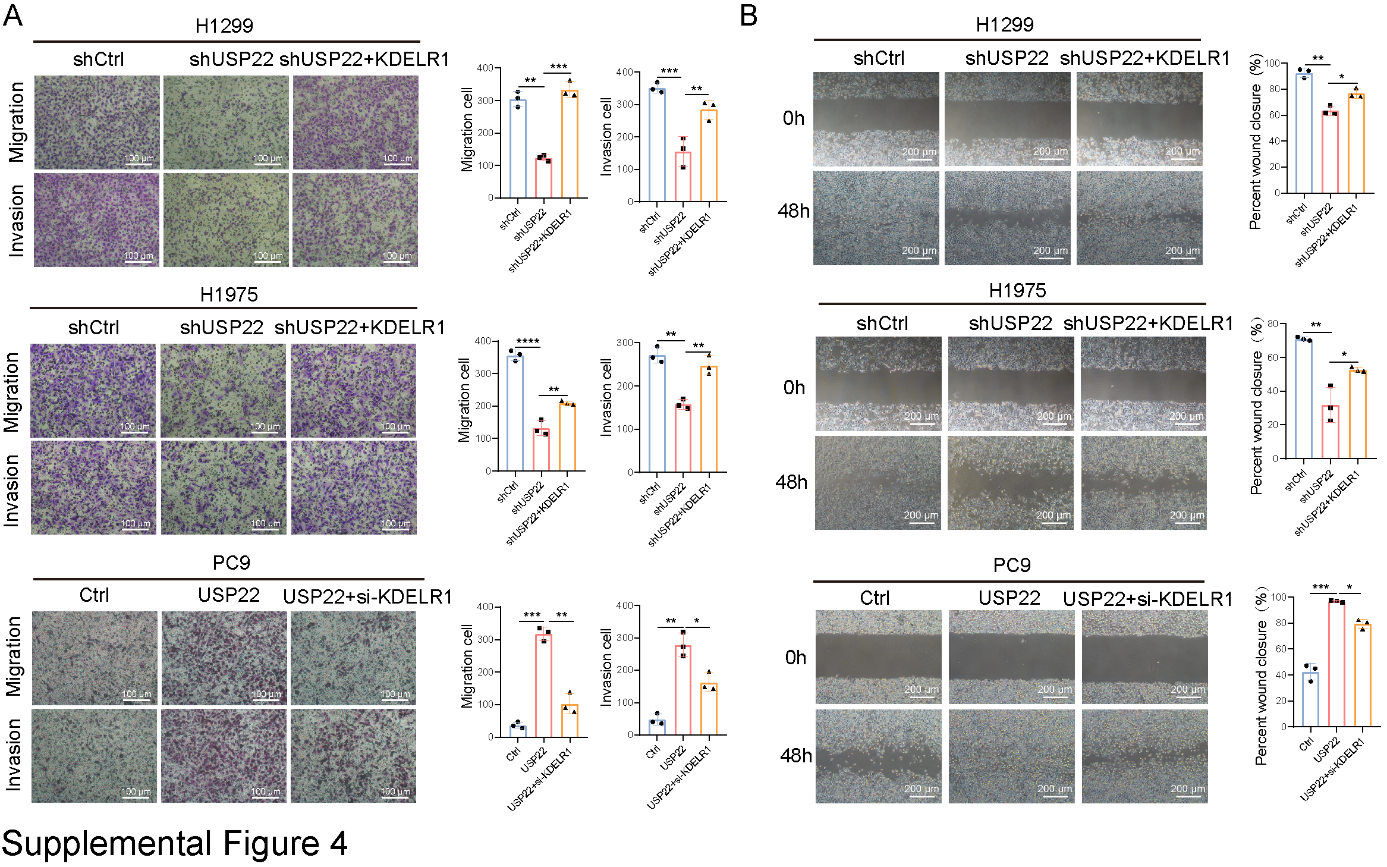
**

**Figure S4. USP22 enhances tumor metastasis by stabilizing KDELR1.** (**A, B**) Transwell and wound healing assays were performed on H1299 and H1975 cells transfected with shCtrl and shUSP22, and reconstituted with KDELR1 plasmid; PC9 cells transfected with Ctrl and USP22, and reconstituted with KDELR1 siRNA. Scale bars, 100 μm (**A**). Scale bars, 200 μm (**B**). Data are presented as mean ± SD. Statistical significance was determined by ANOVA test. **p* < 0.05, ***p* < 0.01, ****p* < 0.001, *****p* < 0.0001.


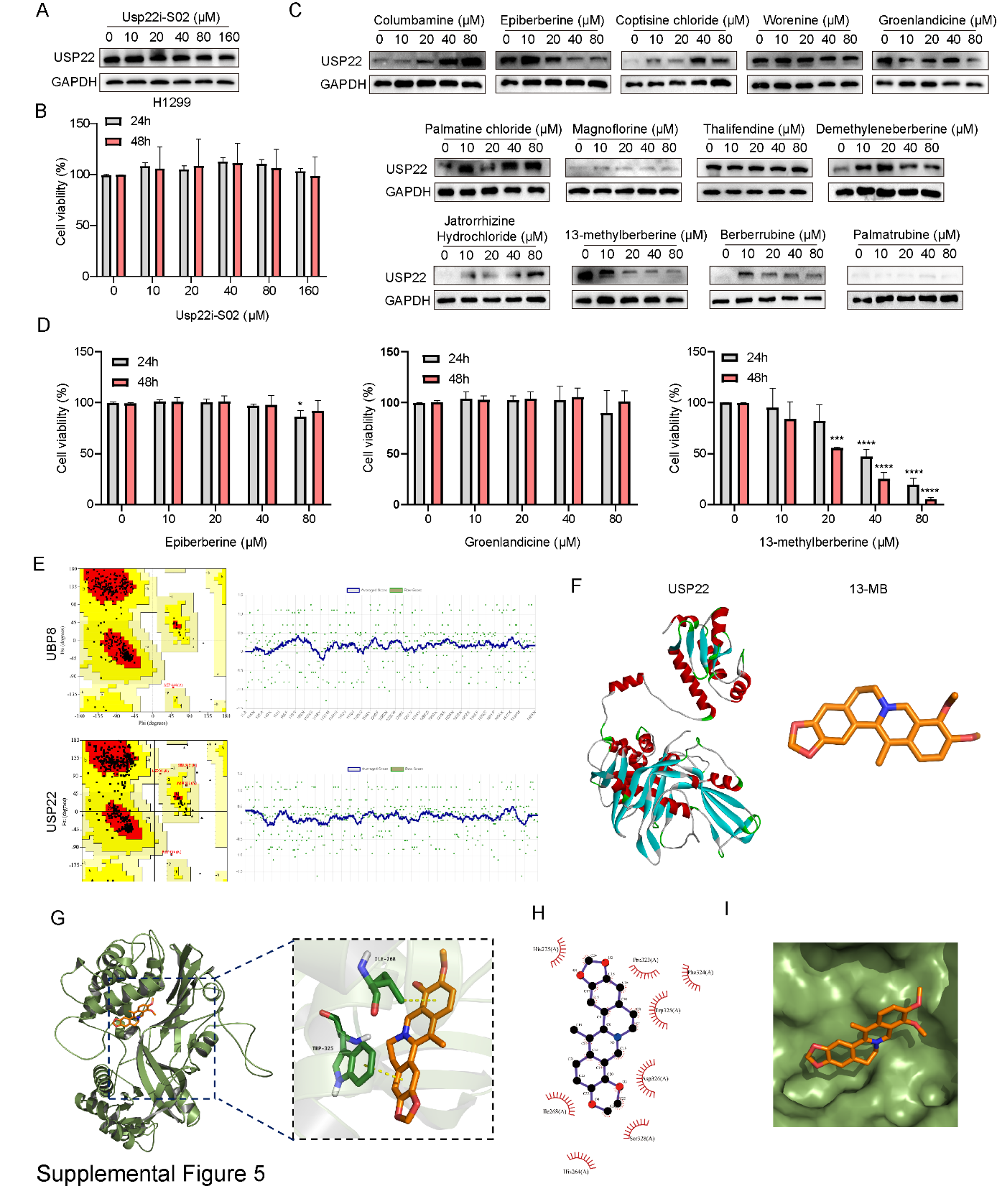


**Figure S5. Screening of inhibitors targeting USP22.** (**A**) Inhibitory effect of Usp22i-S02 on USP22 expression in H1299 cells. (**B**) Effect of Usp22i-S02 on H1299 cell viability as detected by CCK-8 assay. (**C**) Effects of different compounds at different concentrations on USP22 expression. (**D**) Effects of different compounds at different concentrations and times on H1299 cell viability. After treatment with the compounds for 24 and 48 h, cell viability was examined using the CCK-8 assay. (**E**) Conformation of USP22 detected by the Ramachandran plot and 3D conformation evaluation map is reasonable. UBP8 structure (PDB: 3MHS) was selected as the template protein to construct the USP22 model using the Swiss Model. 3MHS (top), USP22 (bottom). (**F**) 3D conformation of USPP22 by homologous model (left panel). Schematic representation of the 2D plane of 13-MB (right panel). (**G**) A diagram illustrating the docking of 13-MB with USP22. USP22 is shown as a cartoon, and 13-MB is shown as a yellow stick. (**H**) Ligplot showing the contact between USP22 and 13-MB. (**I**) Surface representation of the binding pocket of 13-MB to USP22. Data are presented as mean ± SD. Statistical significance was determined by ANOVA test. **p* < 0.05, ***p* < 0.01, ****p* < 0.001, *****p* < 0.0001.
