# Supplementary material for: Ubiquitin‐Specific Protease 22 Plays a Key Role in Increasing Extracellular Vesicle Secretion and Regulating Cell Motility of Lung Adenocarcinoma
Source: Adv Sci (Weinh). 2024 Aug 5;11(38):2405731. doi: 10.1002/advs.202405731 (PMC11481270; doi:10.1002/advs.202405731)
Supplement: Supplementary file 2 — Supporting Information [file ADVS-11-2405731-s002.zip › Table 1.docx]

**Supplemental Table 1: Chemical name and structure of berberine analogs**

| Chemical name | Chemical structure | Efficacy in USP22 inhibition |
| --- | --- | --- |
| Columbamine | 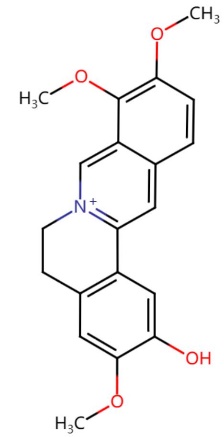 | - |
| Epiberberine | 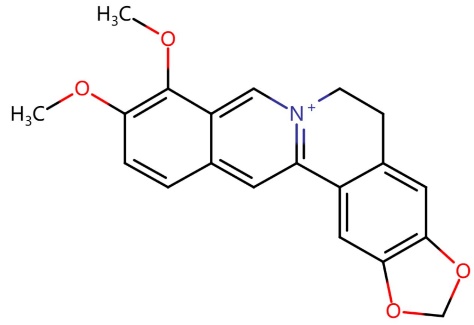 | + |
| Coptisine chloride | 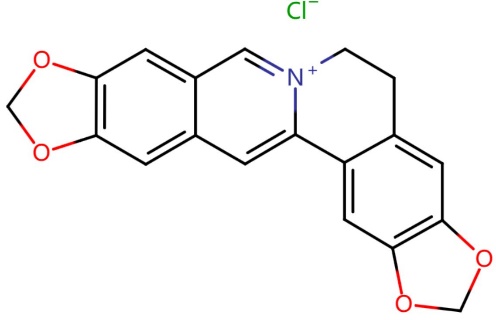 | - |
| Worenine | 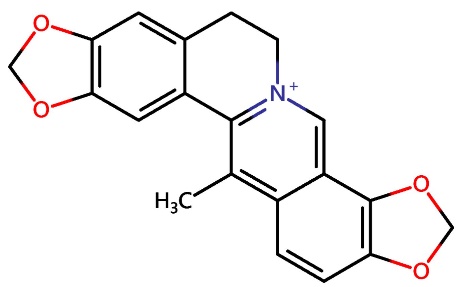 | - |
| Groenlandicine | 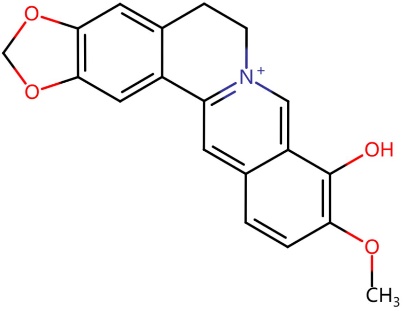 | + |
| Palmatine chloride | 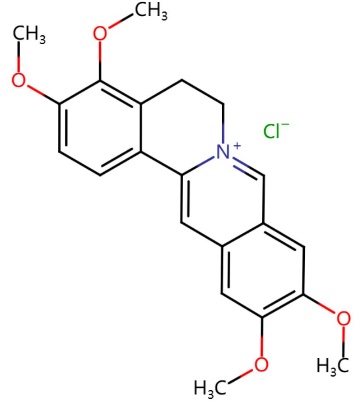 | - |
| Magnoflorine | 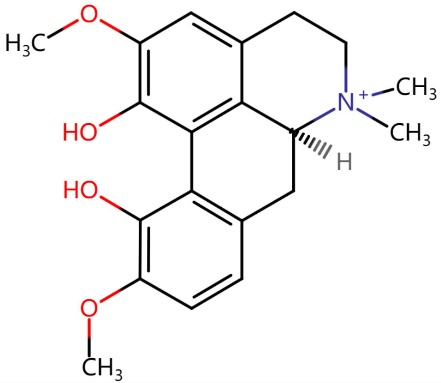 | - |
| Thalifendine | 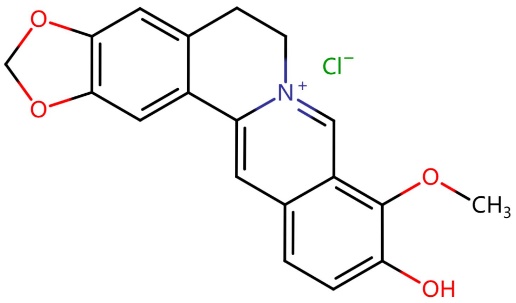 | - |
| Demethyleneberberine | 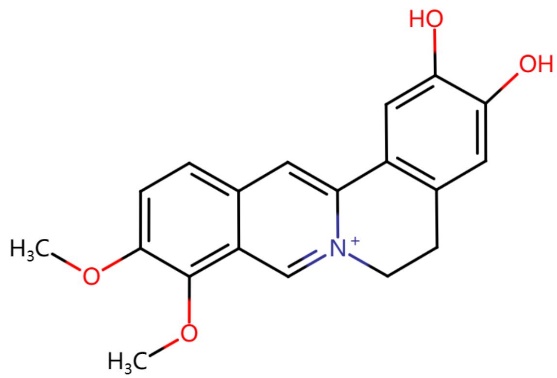 | - |
| Jatrorrhizine Hydrochloride | 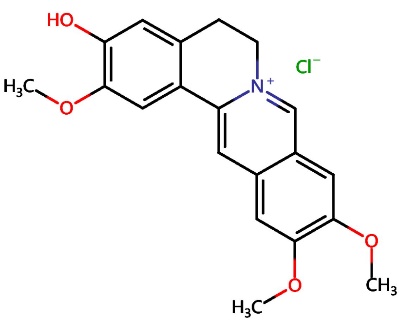 | - |
| 13-methylberberine | 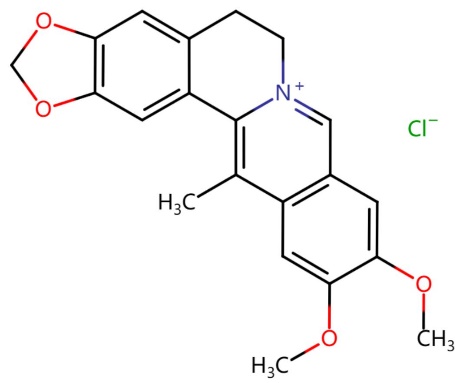 | + |
| Berberrubine | 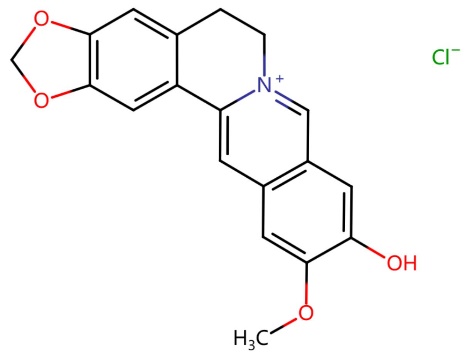 | - |
| Palmatrubine | 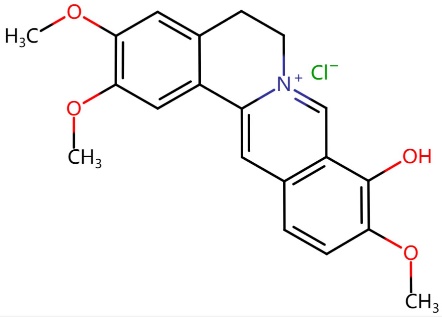 | - |
